# Telecare for Diabetes, CHF or COPD: Effect on Quality of Life, Hospital Use and Costs. A Randomised Controlled Trial and Qualitative Evaluation

**DOI:** 10.1371/journal.pone.0116188

**Published:** 2015-03-13

**Authors:** Timothy W. Kenealy, Matthew J. G. Parsons, A. Paul B. Rouse, Robert N. Doughty, Nicolette F. Sheridan, Jennifer K. Harré Hindmarsh, Sarah C. Masson, Harry H. Rea

**Affiliations:** 1 Department of General Practice and Primary Health Care, University of Auckland, Auckland, New Zealand; 2 Faculty of Medical and Health Sciences, The University of Auckland, Auckland, New Zealand, in partnership with Waikato District Health Board, Waikato, New Zealand; 3 Department of Accounting and Finance, The University of Auckland Business School, The University of Auckland, Auckland, New Zealand; 4 Department of Medicine, University of Auckland and Greenlane Cardiovascular Service, Auckland City Hospital, Auckland, New Zealand; 5 School of Nursing, Faculty of Medical and Health Sciences, University of Auckland, Auckland, New Zealand; 6 Ngati Porou Hauora Charitable Trust, Te Puia Springs, Tairawhiti/East Coast, New Zealand; 7 Department of Medicine, University of Auckland, Auckland, New Zealand; Weill Cornell Medical College Qatar, QATAR

## Abstract

**Objectives:**

To assess the effect of telecare on health related quality of life, self-care, hospital use, costs and the experiences of patients, informal carers and health care professionals.

**Methods:**

Patients were randomly assigned either to usual care or to additionally entering their data into a commercially-available electronic device that uploaded data once a day to a nurse-led monitoring station. Patients had congestive heart failure (Site A), chronic obstructive pulmonary disease (Site B), or any long-term condition, mostly diabetes (Site C). Site C contributed only intervention patients – they considered a usual care option to be unethical. The study took place in New Zealand between September 2010 and February 2012, and lasted 3 to 6 months for each patient. The primary outcome was health-related quality of life (SF36). Data on experiences were collected by individual and group interviews and by questionnaire.

**Results:**

There were 171 patients (98 intervention, 73 control). Quality of life, self-efficacy and disease-specific measures did not change significantly, while anxiety and depression both decreased significantly with the intervention. Hospital admissions, days in hospital, emergency department visits, outpatient visits and costs did not differ significantly between the groups. Patients at all sites were universally positive. Many felt safer and more cared-for, and said that they and their family had learned more about managing their condition. Staff could all see potential benefits of telecare, and, after some initial technical problems, many staff felt that telecare enabled them to effectively monitor more patients.

**Conclusions:**

Strongly positive patient and staff experiences and attitudes complement and contrast with small or non-significant quantitative changes. Telecare led to patients and families taking a more active role in self-management. It is likely that subgroups of patients benefitted in ways that were not measured or visible within the quantitative data, especially feelings of safety and being cared-for.

**Trial Registration:**

Australian New Zealand Clinical Trials Registry ACTRN12610000269033

## Introduction

Globally, 60 to 75 percent of all deaths are due to chronic conditions, and the proportion is rapidly increasing along with the associated cost and demands on workforce [1]. Chronic conditions are the leading cause of unequal health outcomes across social groups [2, 3]. “Telecare”, as used here, is the use of electronic information and telecommunications technologies to support remote 24 hour and asynchronous clinical health care. Studies of the effectiveness of telecare are promising but give mixed results, suggesting that there is still much to learn about fitting telecare into complex health systems [4–6]. Questions remain about whether telecare will improve patient outcomes, decrease use of resources, and be acceptable to providers and consumers across multiple cultures and across multiple health care systems.

The aims of this study were: to assess the effect of telecare on health related quality of life and associated measures; to assess the effect of telecare on hospital admissions, length of stay, emergency department and outpatient visits, and costs; and to investigate acceptability of telecare to patients, caregivers and health care professionals. We chose three of the commonest chronic conditions, chronic obstructive pulmonary disease (COPD), congestive heart failure (CHF) and diabetes. We conceptualised telecare as being more than just technology, rather that the technology was one component of a system of care. We considered that if telecare enhanced patient self care this would be desirable in itself and it would be more likely to reduce health services workload and justify ongoing funding support. We considered that seeing daily data might help both patients and their clinicians make better decisions sooner which could lead to improved wellbeing for patients and decreased use of emergency department and hospital admissions. Quantitative and qualitative data gave complementary and contrasting views of the study results so we considered it necessary to present both together.

## Methods

The protocol for this trial and supporting CONSORT checklist are available as supporting information; see S1 CONSORT Checklist and S1 Protocol.

Our rationale for combining conditions (CHF, COPD and diabetes) rests on four main arguments and a statistical justification. First, patients usually have more than one condition. Multi-morbidity and complex interactions between conditions are the norm. For example, in the general population in Scotland the average person had 4 or more long term conditions by age 70 [7]. Second, patients are distressed by physical and especially psychological symptoms which are not specific to disease labels, and we believe that degrees and mechanisms of distress have more in common than not across the range of chronic conditions. Thus our main outcome measures were quality of life, anxiety and depression, self efficacy, and qualitative indicators of satisfaction and distress. Third, our approach is aligned with an emphasis on person-centred care, relationship-centred care, a shift to functional assessment and goal-based care, and self-management strategies that foster generic rather than disease-specific skills [8]. Our intervention was premised, in part, on teaching patients and carers to improve their self-management based on data they collected and entered into the telemonitoring system. Fourth, models of health system organisation designed to respond to chronic conditions are generic rather than disease-specific, as in the Chronic Care Model and primary health care [9, 10]. Our interest is in extending services beyond the current single-disease focus underlying many health services. “In view of the high degree of comorbidity, even in a nonelderly population, single-disease management does not appear promising as a strategy to care for patients” [11]. Finally, our decision to combine medical conditions—one at each site—is statistically justified. Using site as a fixed effect in multilevel regressions allowed us to both remove the effect of site from other measurements and to detect an independent effect of site.

The quantitative report of the randomised controlled trial follows the CONSORT statement as extended for non-pharmacologic interventions [12, 13]. The trial was registered with the Australian New Zealand Clinical Trials Registry, reference ACTRN12610000269033. All pre-planned outcomes are reported here. The qualitative results are reported following the COREQ checklist for the domains of study design and data analysis and reporting except that we have provided more limited data on the research team and reflexivity [14].

### Ethics statement

Written consent was obtained from all participants. All participants were adults. (The youngest participant was 26 years old.) Ethics permission was granted by the New Zealand Ministry of Health Multi-region Ethics Committee, reference MEC/10/04/031.

### Participants

Patients were eligible if they were adults (in our system this means aged 16 or over), lived at home, were able to communicate in English (or had someone in their household able to translate), and were able to physically manage the equipment (or had someone available to assist). Exclusion criteria were significant cognitive impairment (less than 8 out of 10 on the Abbreviated Mental Test Score), serious current physical or mental illness, or had previously used telecare.

The trial was conducted in three centres: Site A is a major city hospital and contributed patients with congestive heart failure (CHF). It had an established secondary care multidisciplinary CHF service with experience in an earlier telecare pilot project. Site B is a major city hospital and contributed patients with chronic obstructive pulmonary disease (COPD). It had an established secondary care multidisciplinary COPD service. Site C is a remote primary care site in a rural settlement one hour away from a regional city, in an impoverished area with a high proportion of indigenous Māori. Patients nearly all had type 2 diabetes and most had other long-term conditions. Site C contributed only intervention patients, as the area is under-served and they wanted to use the opportunity to increase services for their patients and considered it unethical to enrol their patients into a control group. The centres were chosen on the basis of established relationships with the investigators and known capacity and capability, as well as the need to test the system across a range of health care services and telephone systems.

At Sites A and B potentially eligible patients were identified by a clinical nurse specialist as they came into the hospital for usual care relating to their condition. If patients were willing, this nurse passed their details to the independent research nurse who met with them and undertook the formal consenting process. At Site C, potential participants were identified jointly by the practice nurse and general practitioner, based on clinic records. The practice nurse then contacted the patient and undertook the formal consenting process then forwarded their details to the research nurse. At each site the research nurse was independent of the team providing ongoing clinical care. Unfortunately the clinicians screening their patients for those who were eligible did not keep sufficient records of their screening process to provide a reliable count of people who were eligible but did not enter the trial.

### Intervention

Intervention patients were provided with a ‘health hub’ supplied by Docobo (www.docobo.co.uk). The hub was a small device with a LCD display to provide instructions, ask pre-programmed disease-specific questions, or convey short messages from the nurses monitoring the data. Patients entered data manually using buttons on the hub. Patients with CHF were provided with electronic weighing scales, a blood pressure monitor and a pulse oximeter. Patients with COPD were provided with scales and a pulse oximeter. Patients with diabetes were provided with scales, a glucometer, a blood pressure monitor and a pulse oximeter. We chose to have patients enter data manually because we considered this process was more likely to engage them in self-care decisions than if the data were collected automatically. The hubs were connected by telephone land-lines rather than by wireless or cellular technology to reduce costs and because some of the rural areas had no cellular coverage at the time of the study. Data were collected by Docobo and relayed to the monitoring stations where they were viewed on a proprietary web-based interface.

Each intervention patient was initially clinically reviewed either in clinic by a cardiologist (Site A), by a respiratory nurse specialist in the patient’s home (Site B) or at the primary care clinic by the primary care doctor (Site C). At initial review individualised limits were set for each monitored parameter. Following this, patients were visited at home by nurses who set up the telecare equipment and trained patients, and family members where relevant, to use it. Each of these nurses had received at least four hours training from Docobo after which they were supported by the project manager. Technical backup was provided by the project manager and a Docobo technician. Data were routinely collected once a day, usually in the morning, and transmitted in batches to Docobo at midnight, for review by nurses the following weekday morning. There was provision for patients to send additional data when required, such as if the monitoring nurse contacted them and requested additional measurements. At the monitoring stations, the nurses would see summary information from each patient on a single screen, annotated with red, yellow or green indicating whether readings were within targets set for that patient. Black indicated that no data had been received. Nurses were expected to use the system to record their response to abnormal or absent results.

Control patients received usual care. In Site A and Site B this meant they were seen when in hospital or at outpatients by the same team who provided telecare. At Site A, patients in both intervention and control groups were taught to self-care using a detailed tool (www.heartfoundation.org.nz/programmes-resources/health-professionals/heart-failure-tool/information-for-patients). At Site B there was no formal tool but patients in both groups were often followed by telephone and/or by home visit after leaving hospital. All Site C patients were in the intervention group, but before and after the intervention patients were under usual care. The primary care nurse was not involved in their hospital inpatient or outpatient care, but would routinely contact them and might visit them at home after a hospital admission.

The health professionals at Site A consisted of one clinical-academic cardiologist with sub-speciality expertise in heart failure and three heart failure nurse specialists. At Site B there were three respiratory physicians, two respiratory nurse specialists and one respiratory nurse practitioner who had prescribing rights. In Site C there were two consecutive experienced general practitioners, a practice nurse, a rural health nurse and a kaiawhina (community health worker).

The ‘output’ of the intervention was two-fold—at patient level and clinician-level. Patients (and potentially their family) generated regular information which they entered into the telecare system, giving them the opportunity to make regular self-management decisions. Clinicians received regular information which might otherwise have remained unknown to them. There were no set guidelines provided for clinicians to follow in the intervention other than to provide best practice care based on the review of the their (near) daily feedback via telecare. The advice given to patients was not different from than given if the clinician had seen the patient in a face to face consultation, however the clinicians were able to initiate a phone call or other contact. Clinicians kept a log of activities such as phone calls, home visits and patient clinic visits, reported in Table 1, but did not record the content of their consultations.

**Table 1:** Cost of nursing service provision for the trial, based on nurse log records of activities and times.

	Item Count	Total Cost		Product Sustaining	Batch	Unit
Install equipment, education, consent, set up alerts, medical history	225	5,401	13.3%		5,401	
Routine daily data review	4465	11,152	27.4%			11,152
Alert review	296	703	1.7%			703
Liaise with specialist	61	285	0.7%		285	
Telephone call with patient	1070	2,525	6.2%			2,525
Telephone call with GP	29	94	0.2%			94
Telephone call with Practice Nurse	182	878	2.2%			878
Clinic visit	428	4,599	11.3%			4,599
Home visit	587	8,986	22.1%			8,986
Telecare equipment issues	188	1,647	4.0%	1,647		
Liaison with project co-ordinator	32	302	0.7%	302		
Other	333	3,977	9.8%			3,977
Review with specialist	18	146	0.4%		146	
**Subtotal of Nursing Related Costs**		*40,695*	*100.0%*	*1,949*	*5,831*	*32,914*
Nursing Related Costs		40,695	47.8%	1,949	5,831	32,914
Training for nursing staff		3,158	3.7%	3,158		
Internet communication costs (fee charged by provider)		41,262	48.5%		41,262	
**Total costs of providing service**		**85,115**	**100.0%**	**5,107**	**47,093**	**32,914**
Equipment purchase		**93,474**				

Numbers are New Zealand dollars. Cost per item based on average time taken and hourly rate of nurses involved.

### Data collection

#### Patient and caregiver questionnaires

Data collection at baseline, 3 months and 6 months was undertaken by a research nurse who was not part of the clinical team. The Short Form 36, the Hospital Anxiety and Depression and the Self Efficacy for Managing Chronic Disease scales were administered at each visit. The Short Form 36 was given to the principle informal carer at baseline, 3 months and 6 months with a request to self-complete. Informal carers were mostly unpaid family members. Their return rate was poor and the data are not reported here. Patient baseline questionnaires also asked about demographics, social and economic circumstances and functional disabilities for vision and hearing. At 3 months and 6 months intervention patients were asked about their experiences of telecare.

#### Short Form 36 survey tool version 1 (SF-36)

The SF-36 health survey uses 36 questions to explore eight health concepts which can be collated into physical and mental component summary scores. The instrument has been repeatedly used in New Zealand and is one of the most widely used measures of health related quality of life in the world. Internal consistency alpha of scales exceeds 0.8 across large studies and extensive psychometric analyses are available [15]. The scores are standardised to fall between 0 and 100 where a high score is good. Expert consensus has judged that for asthma, COPD and heart disease in the United States, the Minimum Important Difference is about 10 [16].

#### Hospital Anxiety and Depression (HAD) [17]

This instrument has two scales, anxiety (7 items) and depression (7 items). Each item is scored 0 to 3 so that the maximum is 21 for anxiety and 21 for depression where 8 or more is considered borderline and 11 or more is considered definite for the condition. It is widely used including in New Zealand, and shows internal consistency coefficients of 0.72 to 0.93 across both scales and a wide range of acute and chronic patients and the general population [15].

#### Self-Efficacy for Managing Chronic Disease [8]

This scale covers domains that are common across many chronic diseases—symptom control, role function, emotional functioning and communicating with physicians. The instrument has six items, each scored 1–10, and a higher score is higher self-efficacy. The Stanford Patient Education Research Centre (the originators of this scale, http://patienteducation.stanford.edu/research/secd6.html) reports psychometric properties from a study of 605 United States patients with mixed long term conditions, showing that across 6 items there was an observed range was 1–10, mean 5.17, SD 2.22 and internal consistency reliability 0.91.

#### Self Care of Heart Failure Index (SCHFI) version 6.2 [18, 19]

The scale has 23 items designed to measure the effects of heart failure and treatments for heart failure on three domains—maintenance, management and confidence. Each domain is standardised to score between 0 and 100, where a high score is good and a score above 70 indicates adequate self-care although benefit occurs at lower levels [19]. A change score of more than 0.5 standard deviation is considered clinically relevant [19]. Psychometric testing on 154 CHF patients in the United States showed that reliability of the maintenance subscale was lower than desired (alpha .55) but this was expected because the items reflect behaviours known to vary between individuals. Reliability of the confidence scale was adequate (alpha 0.82). The management scale was unchanged from the original instrument where it had alpha 0.72 on 760 patients [18].

#### The St George Respiratory Questionnaire for COPD (SGRQ-C) [20, 21]

The questionnaire contains 40 items in three components: symptoms, activity, and impacts. Scores range from 0 to 100 where down is good. The Minimum Important Difference is 4. The COPD-specific version removed poorly performing items from the original scale after a Rasch analysis of data on 893 patients. The person separation index (analogous to Cronbach’s alpha) was 0.74 for symptom items, 0.88 for activity items and 0.84 for impact items. The questionnaire has been widely used including in New Zealand.

#### Summary of Diabetes Self-Care Activities [22]

This scale collects self-report assessments of: general diet, specific diet, exercise, blood-glucose testing, foot care, and smoking. Except for smoking, the results are reported for each domain as number of days per week a desirable activity was followed. Psychometric data from 1988 patients in 7 studies showed average inter-item correlations within scales were high (mean 0.47), with the exception of specific diet; and test-retest correlations were moderate (mean 0.40) [22]. It has been translated into at least two non-English languages and used with at least four ethnic groups in several countries but there are no published reports using it in New Zealand.

### Health service use and costs

Counts of hospital admissions, length of stay, emergency department attendance and outpatient visits were extracted directly from the hospital management records. For reporting and management purposes, hospital use is classified as either emergency department (if the visit is less than 3 hours and the patient leaves) or a hospital admission (if the visit is 3 hours or longer, whether or not they are admitted to an overnight hospital bed). The cost analysis used hospital data for inpatients and outpatient visits to estimate cost savings. When considering potential cost savings, there is a strong case for using direct costs rather than total costs, as the latter include indirect allocations for facility wide costs such as corporate and occupancy. We recognise that ‘savings’ on direct costs may simply be used to provide other services, however, direct costs remain a better indicator of potential cost behaviour for comparative purposes. All costs are given in New Zealand dollars. At the time the study was done one NZ dollar was worth approximately 0.8 US dollars.

Nurses working with the intervention patients kept a log of their activities specific to telecare. Nurse costs were estimated from these time logs together with payroll data and related costs being the payroll related costs such as pension funds and government levies. These were categorized in terms of an activity based costing hierarchy into product sustaining, batch and unit levels (the fourth level, facility costs, were not included in this analysis). Unit level costs are those that vary directly with interactions with patients whereas batch level costs are those that relate more to managing the service such as the initial set up of equipment in the patient’s home which are not affected by the number of subsequent visits. Product sustaining costs are those that relate to providing the service and in this setting include the training and dealing with the equipment suppliers. Note that the drivers of cost vary depending upon the level of the cost hierarchy [23].

All patients were asked to keep a log of number of general practice consultations and associated costs included fees paid to the practice, travel time and distance, and pharmacy.

### Interviews, focus groups and questionnaires

Interviews and focus groups were conducted with all the relevant clinicians and managers in each service during the trial to gain early feedback and make minor adaptations to the system if needed. After the trial, interviews and focus groups with the practitioners and service managers explored attitudes to telecare and the specific system used. Some 27 clinicians and service managers contributed to at least one interview or focus group. Interviews with eight patients during the trial focussed on usability of the technology. After the trial, two focus groups with a total of twelve patients at Site C sought feedback and suggestions for the future. All participants at this site were invited. In addition, open and closed questions in the questionnaires at 3 months and 6 months invited comments on experiences of telecare and related attitudes of every intervention patient.

### Statistical analysis

Sample size calculations, using a power of 90% and alpha 0.05, were based on several scenarios. Local data on an frail elderly population of 205 people found a mean total SF36 score of 58.5 (SD 18.65) and we calculated power to detect a change to mean 68.5 (SD 18.65) [24]. Another trial measured SF36 physical score in 280 patients with advanced heart failure [25]. From these data we calculated power to detect a change from mean 32 (SD 8.7) to 42 (SD 11.4). According to these calculations we needed a total of 148 patients and 44 respectively (at 1:1 intervention to control). Calculations based on hospital admissions, length of stay and cost literature suggested sample sizes that ranged from 100 to 5000. We estimated that about 20% would drop out or be lost to follow up. For COPD and heart failure, we estimated that the death rate would be about 20% per year. We made a pragmatic decision, following discussions with the clinicians at the trial sites, to target recruiting a minimum of 75 patients for each of intervention and control, knowing that we would be underpowered to detect change on some of the clinical scores, but nevertheless expecting to gain an estimate of effect size.

Random sequences were generated by TK in Excel in blocks of eight within each site and recorded on paper slips within opaque, numbered envelopes. Allocation ratio was one treatment to one control. The study nurse at each site obtained consent, recorded patient identification details then opened the envelopes in sequence, recording envelope number for each patient. Once allocation was assigned, neither patients nor their health professionals were blind to the intervention. Blind adjudication over whether hospital admissions and outpatient appointments were relevant to COPD was conducted by TK and HR. It is unusual to have one site contributing only intervention patients. We justify this by our assertion that people with different disease labels nevertheless have more in common than different with respect to their experience and self-management of chronic disease. The statistical testing is sensitive to both changes in individuals over time (all groups) and between intervention (measured in all groups) and control (measured in groups A and B). The primary analysis includes all site but sensitivity tests repeat the analyses with site C excluded.

SF36 questions were initially analysed using software provided by QualityMetric Incorporated (the license holders for SF36), which produced standardised scores for each patient at each time point. These scores were used in subsequent analyses. All analyses were on an intention-to-treat basis, including all patients at all time-points where data were available. For psychometric outcomes, withdrawals and deaths are treated as missing data. For count data, adjustment for exposure (detailed below) effectively adjusted for withdrawals and deaths. There was no imputation of missing data. All analyses were conducted in Stata 12.1 (College Station, Texas). Continuous variables were analysed using mixed effects models that included site as a fixed effect and patient as a random effect. Sensitivity analyses excluded Site C and adjusted for age and gender. The main outcome for the psychometric scales was assessed from the interaction between intervention group and time. Mixed-effects negative binomial regressions were used to model count data including numbers of hospital attendance, length of stay and costs in analyses were adjusted for patient age, gender, ethnicity, and site as a factor variable. We rejected the alternative of estimating robust confidence intervals based on clustering by site because we selected sites by availability rather than sampling, and because, in this data, the analysis using site as a factor variable was more conservative. Exposure, for hospital use, was measured by days alive and not in hospital. Exposure, for total days in hospital, was measured by days alive. Results were considered statistically significant when p ≤ 0.05.

### Qualitative analysis

Interviews and focus groups were audio-recorded, transcribed verbatim, and imported into NVivo 8 to support a qualitative descriptive thematic analysis [26, 27]. Themes from staff interviews were fed back to them for confirmation and further responses. Themes from Site C interviews were taken back to a focus group at Site C to confirm their perspectives had been correctly interpreted. The open questions from the patient questionnaires were explored using descriptive content analysis. Themes are reported with quotes considered to be typical unless explicitly noted. Only brief comments from the qualitative analysis are presented here.

## Results

**Fig 1:**
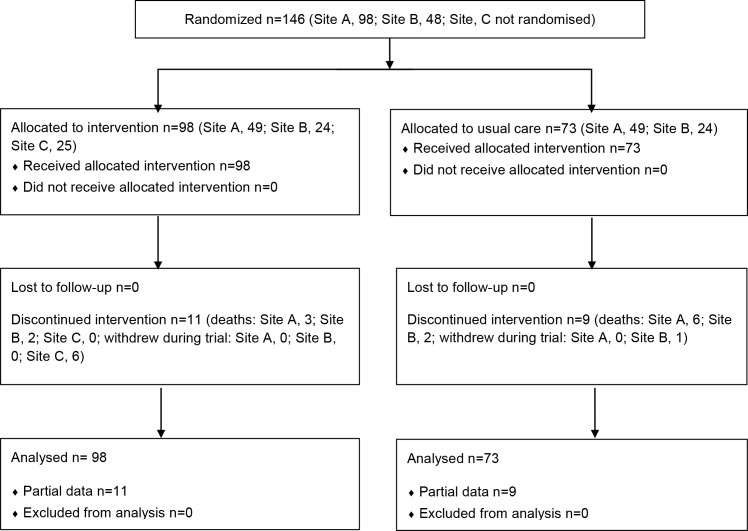
Trial numbers flow diagram.

Recruitment ran from September 2010 to August 2011. A total of 98 patients entered the intervention, 73 entered the control group, and all contributed to at least the hospital outcome data. Nine patients died during the trial. Flow of participants is shown in Fig. 1.

Baseline characteristics of participants are shown in Table 2. The groups appear well matched apart from the telecare group including more Māori (from Site C), and at Site A, the intervention group had more hospital admissions and more days in hospital in the previous year than the control group. At Site A, more than a quarter of participants had severe CHF, and at Site B most had severe COPD.

**Table 2:** Baseline demographic and clinical characteristics of participants by intervention group.

		Telecare	Usual care
**Site A**		**49**	**49**
	Age, years	72 (62–83)	72 (60–77)
	Female	19 (39%)	14 (29%)
	*Ethnicity*		
	NZ European / Other	40 (82%)	37 (76%)
	Māori	3 (6%)	3 (6%)
	Pacific	6 (12%)	9 (18%)
	*Housing*		
	Own / family member’s home	32 (65%)	34 (69%)
	Rent	15 (30%)	12 (24%)
	Retirement village or similar	2 (4%)	3 (6%)
	Highest education qualification: primary school	9 (18%)	7 (14%)
	*Personal income in last year*		
	≤ $20,000	26 (53%)	21 (43%)
	No response	7 (14%)	15 (31%)
	Vision: moderate or worse difficulty reading book or newspaper	9 (18%)	3 (6%)
	Hearing: moderate or worse difficulty in conversation with one person in a quiet room	3 (6%)	0 (0%)
	New York Heart Association class 3 or 4	19 (5 unknown)	15 (9 unknown)
	Left Ventricular ejection fraction < 30%	15 (2 unknown)	13 (9 unknown)
	Hospital admissions in previous year	12	79
	Days stay in hospital in previous year	866	553
	*Numbers with (all had CHF):*		
	Diabetes	16 (0 unknown)	16 (6 unknown)
	Chronic respiratory disease	15 (I unknown)	9 (7 unknown)
**Site B**		**24**	**24**
	Age, years	67 (64–74)	67.5 (63–72.5)
	Female	9 (38%)	9 (38%)
	*Ethnicity*		
	NZ European / Other	18 (75%)	16 (67%)
	Māori	1 (4%)	3 (13%)
	Pacific	5 (21%)	5 (21%)
	*Housing*		
	Own / family member’s home	17 (71%)	15 (63%)
	Rent	6 (25%)	9 (38%)
	Retirement village or similar	1 (4%)	0 (0%)
	Highest education qualification: primary school	4 (17%)	2 (8%)
	*Personal income in last year*		
	≤ $20,000	14 (58%)	14 (58%)
	No response	5 (21%)	5 (21%)
	Vision: moderate or worse difficulty reading book or newspaper	3 (13%)	3 (13%)
	Hearing: moderate or worse difficulty in conversation with one person in a quiet room	3 (13%)	1 (4%)
	Resting oxygen saturation ≤ 90% on air	6	2
	Global Initiative for Chronic Obstructive Lung: Disease classification severe or very severe [38]	18	21
	Hospital admissions in previous year	71	63
	Days stay in hospital in previous year	199	210
	*Numbers with (all had COPD):*		
	Diabetes	4	2
	CHF	5	5
**Site C**		**25**	**0**
	Age, years	57 (53–60)	-
	Female	15 (60%)	-
	*Ethnicity*		
	NZ European / Other	3 (12%)	-
	Māori	22 (88%)	-
	Pacific	0 (0%)	-
	*Housing*		
	Own / family member’s home	20 (80%)	-
	Rent	5 (20%)	-
	Retirement village or similar	0 (0%)	-
	Highest education qualification: primary school	6 (24%)	-
	*Personal income in last year*		
	≤ $20,000	3 (12%)	-
	No response	14 (58%)	-
	Vision: moderate or worse difficulty reading book or newspaper	4 (16%)	-
	Hearing: moderate or worse difficulty in conversation with one person in a quiet room	3 (12%)	-
	Hospital admissions in previous year	13	-
	Days stay in hospital in previous year	72	-
	*Numbers with:*		
	Diabetes	22	-
	CHF	6	-
	COPD	4	-

Results are numbers, or median (interquartile range), mean (SD) or number (%).

Table 3 summarises the results of SF36, HAD and SEMCD scores from the three sites, and disease-specific results from each site. No significant change over time was seen in SF36 or SEMCD results or disease-specific scores. HAD scores for anxiety and depression both improved significantly although the improvement was smaller than our assumed minimal important difference. The decrease in HAD depression score was not significant if Site C was excluded. Otherwise exclusion of Site C, and adjustment for age and gender, made no substantive difference to any result.

**Table 3:** Baseline and 6 month scores on measurement scales.

		Telecare	Usual care	Statistics
*Short Form 36*				
Physical component score	Baseline	36.2 (10.5)	37.7 (10.4)	0.32, p 0.67;	0.35, p 0.67;	0.30, p 0.69
	6 months	37.7 (11.0)	38.8 (11.6)	
Mental component score	Baseline	48.5 (11.9)	49.2 (11.6)	-0.46, p 0.64;	0.47, p 0.63;	0.21, p 0.85
	6 months	49.1 (12.1)	50.7 (11.2)	
*Hospital Anxiety and Depression*				
Anxiety	Baseline	4.1 (3.1)	3.5 (2.8)	-0.51, p 0.02;	0.50, p 0.02;	0.50, p 0.04
	6 months	3.9 (3.1)	4.4 (3.4)	
Depression	Baseline	4.6 (3.4)	3.7 (3.1)	-0.49, p 0.04;	0.49, p 0.04;	0.39, p 0.13
	6 months	4.2 (3.7)	4.2 (3.2)	
*Self Efficacy for Managing Chronic Disease*	Baseline	6.8 (2.0)	7.0 (1.5)	0.02, p 0.85;	0.03, p 0.84;	0.02, p 0.90
	6 months	6.9 (1.9)	7.0 (1.8)	
*Self Care for Heart Failure Index*				
Maintenance	Baseline	70.2 (12.0)	70.8 (11.8)	0.89, p 0.43;	0.83, p 0.44
	6 months	73.6 (8.6)	72.2 (10.7)	
Management	Baseline	61.6 (16.7)	64.2 (17.6)	0.14, p 0.95;	0.18, p 0.95
	6 months	65.5 (18.5)	70.2 (15.4)	
Confidence	Baseline	67.4 (13.5)	69.8 (13.1)	1.55, p 0.31;	1.64, p 0.28
	6 months	70.9 (13.6)	70.3 (15.9)	
*St George Respiratory Questionnaire*				
Total	Baseline	62.5 (15.1)	60.9 (16.1)	3.29, p 0.12;	3.28, p0.12
	6 months	59.3 (13.5)	51.7 (23.2)	
*Summary of Diabetes Self Care Activities*				
Good diet (days/week)	Baseline	4.4 (1.9)		-0.24 p 0.15;	0.22, p 0.19
	6 months	3.9 (1.6)		
Good exercise (days/week)	Baseline	2.8 (2.5)		0.23 p 0.37;	0.24, p 0.37
	6 months	3.3 (2.3)		
Good sugar testing (days/week)	Baseline	2.8 (2.6)		0.20 p 0.57;	0.21, p 0.55
	6 months	3.6 (3.1)		
Good foot care (days/week)	Baseline	2.9 (2.1)		0.12 p 0.64;	0.12, p 0.63
	6 months	3.3 (2.3)		

Results are mean (SD). Statistics are coefficient of interaction between intervention and time (positive indicates increasing score over time in telecare compared to usual care except diabetes self-care indicates increase score from baseline to six months). The first statistic is the unadjusted model, the second adjusts for age and gender and the third excludes Site C (for measures collected at all Sites). Heart Failure – Site A; Respiratory—Site B; Diabetes—Site C. SF36, Self Efficacy for Managing Chronic Disease, Self Care for Heart Failure Index, Summary of Diabetes Self Care Activities—favourable results higher; Hospital Anxiety and Depression, St George Respiratory Questionnaire—favourable results lower

Most patients indicated they would be prepared to pay for telecare—65/83 (78%) at 3 months, with little change at 6 months (32/43 (74%)). When asked how much they would pay per month (combining 3 and 6 month data), 68/92 (74%) said up to $25, 20/92 (22%) said up to $50 and 4 said up to $100.

Hospital service use and costs are listed in Tables 4 and 5. Compared with usual care, the telecare group showed no significant change in admissions (coefficient 0.32, p 0.15), emergency department visits (coefficient -0.08, p 0.91), outpatient visits (coefficient -3.27, p 0.23), total days in hospital (coefficient 0.51, p 0.09) or total costs of hospital services (coefficient 0.36, p 0.13). Post-hoc contrasts showed costs at Site C were significantly lower than Sites A and B. The overall results for costs did not change following sensitivity analyses; for Site B, we excluded costs for hospital services assessed as not relevant to COPD (the assessment was done by clinicians blinded to trial group allocation); and for Site A we excluded costs for a portion of nurse time that may have been research time mis-attributed to clinical service. Site C hospital use is shown for the period prior to the trial but these data were not used in the calculations presented here.

**Table 4:** Counts of health service use (all causes) and deaths during trial.

		Telecare	Usual Care
Hospital admissions			
	Site A	56	30
	Site B	39	33
	Site C	14	(13)
Hospital days stay			
	Site A	529	214
	Site B	175	155
	Site C	80	(72)
Emergence Department visits			
	Site A	2	5
	Site B	4	1
	Site C	1	(4)
Hospital outpatient visits			
	Site A	330	232
	Site B	139	93
	Site C	25	(86)
Deaths			
	Site A	3	6
	Site B	2	2
	Site C	0	(0)

Usual Care numbers for Site C (shown in brackets) are from the 6 months prior to trial entry. Results are counts.

**Table 5:** Costs per month for hospital services before, during and after trial, New Zealand dollars 2012.

Intervention					
	Pre-trial	Trial	Post-trial	Trial less pre-trial	Post-trial less pre-trial
**Site A**	74,075	78,295	41,237	4,220	-32,838
**Site B**	26,151	15,373	15,950	-10,777	-10,201
**Site C**	5,403	2,863	2,730	-2,539	-2,673
**Total**	**105,628**	**96,531**	**59,916**	**-9,097**	**-45,712**
**Control**					
**Site A**	63,741	47,068	11,358	-16,673	-52,383
**Site B**	15,580	24,990	10,940	9,410	-4,640
**Total**	**79,321**	**72,057**	**22,298**	**-7,264**	**-57,024**

Positive costs in last two columns indicate increased monthly cost, negative indicates decreased costs. Site C patients were all in the intervention group. Costs extracted directly from hospital management system and include all admissions, emergency department visits and secondary care outpatient visits. They do not include primary care or patient costs.

Table 5 compares the monthly costs before, during and after the trial (averaged over 12 months, 6 months and 12 months, respectively). Site A intervention costs increased during the trial and decreased after, while control group costs decreased in both time periods. Site B intervention costs decreased during and after the trial, while control costs increased then decreased. Site C costs decreased in both the trial and post-trial periods.

Total nurse related costs were $40,695 during the trial, shown in Table 1. Sixty percent of the nursing related costs comprise the routine daily data review, clinic visits and home visits. Around 81 percent of the nursing related costs were at the unit level with about 15 percent at batch level. The lower part of the table includes the initial training costs for nursing staff and the communication fees charged by the equipment provider for the trial.

After the trial, Docobo set a communications cost per patient that was not sustainable for the health boards or the primary care provider, so it was rejected. From a health sector perspective, there is a major benefit in exploring whether the telemedicine communications and infrastructure could be either developed internally or purchased to be provided internally. Table 1 shows that the communication costs paid to Docobo were almost half the total ongoing costs during the trial, a rate which was considered too expensive to continue longterm. The final figure in the table is the cost of purchasing the equipment to be used by the patients.

Patient costs at Site A were $4437 in the intervention group and $6564 in the control group, associated with 46 visits and 60 visits to primary care. Corresponding costs for Site B were $9428 (112 visits) and $6950 (85 visits). Site C patient costs during the trial were $2490 but we do not have equivalent costs prior to the trial.

### Qualitative results

We identified patient-related themes of feeling safe, learning and understanding, supporting self-care and family care in ways not achieved with prior care, telecare imposing a routine, technology issues and overall reactions to telecare. Quotes are identified by gender, ethnicity and age when they come from open questions in the questionnaires. These data were generally not available from the focus groups with patients, and health care staff are identified only by role.

The strongest message from patients was that they felt looked after and safe. As one 86 year old European woman, who lived alone and had CHF explained, “*The psychological benefit is hard to explain … It’s magic … it gives me total safety*”. Open questionnaire responses included: “*It monitors me both physically*, *spiritually and mentally … Keeps me safe” (Male*, *Maori*, *54)*.

Patients learned from the feedback gained from taking their own measurements and entering them into the device.

…*it also made me realise that if I had one more prune that night [nurse] was ringing me up the next day to say ‘why was my blood sugars so high*’. … *but it taught me that you can’t eat that many*. *So there was definitely education for me*. *In terms of what food I could eat and what foods I definitely couldn’t eat (Patient*, *focus group 2)*.

There was an acceptable sense of being watched: “*Telecare pulls you up as you’re not the only one that’s seen the results*.” (Patient, focus group 1)

Patients were conscious of paying increased attention to their health—being able to monitor themselves. “*It helps me keep focussed on my well-being” (Female*, *European*, *59)* and “*Keeps me close to the problem—keeps me aware of what is going in the body” (Male*, *European*, *70)*.

…*for me when things go wrong I need to rely on that machine as a monitor because I can’t see inside me*. *But those machines that you’ve given us have given us a better appreciation of where we are at in life (Patient*, *focus group 1)*.

*He loves it*. *He’s really switched onto his stats*. *He’s really good*. *A couple of weeks ago his stats dropped and he phoned and we went over together to see him (Primary care nurse)*.

Staff agreed that telecare helped patients understand their condition and communicate better with health professionals.”*[telecare] is a catalyst for quite rapidly increasing people’s [health] literacy”* (Primary care manager).

Learning still allowed patients to knowingly choose whether or not to alter their behaviour.

*When you see after you’ve taken all your stats and stuff*, *and you write it down in your notebook and have a look and that makes you think*. *‘Oh’ or ‘yay I’m doing something right’*. *Or ‘gee better chop something’*. *So looking at your actual results does make you think ‘mm I need to change’ or ‘oh bugger it I’m not going to change’ (Patient*, *focus group 1)*.

Safety, confidence, learning and self-care were mutually supportive.

*Now I’m confident in myself*, *I’m far more confident in sleeping by myself*. *I know that when I wake up the next morning I’m on the right track*. … *When I hopped on [scales] again and [weight] started going down I thought ‘get the doctor I’m having an hallucination’*. …*I feel proud of myself with those scales*. *I am starting to feel changes within myself (Patient*, *focus group 1)*.

Routine was frequently mentioned as a positive in the open questionnaire responses. *“It’s given me a routine—so I now check my blood sugars more consistently” (Female*, *European*, *57)* was typical. Nevertheless, a small number of patients were pleased to see the devices returned at the end of the trial. One of the open questionnaire responses at 6 months noted: “*Started getting bored using equipment every day” (Female*, *Maori*, *48)*.

The open ended questionnaire item asking “What did you like most about using the equipment?” produced 105 positive responses. We did not find any difference between answers at 3 months and 6 months. Several people noted that it was *“fun”* and/or they felt *“important”*. When asked “What did you like least about using the equipment?” most answers were *“Nothing”* or left blank. Of the 20 other responses, most were about technical difficulties such as with batteries, plugs, telephone lines or power cuts. People who moved location such as for work or holiday had particular difficulties. When we asked what people would miss most when we took the equipment away, again the answers were overwhelmingly positive. *“What*! *The whole set up” (Male*, *European*, *58)* was typical. One interviewed patient summed up her feeling as: *“Giving up the equipment meant that you had given away your insurance*…” (Patient, focus group 2)

Family involvement due to telecare was a recurrent theme. Some patients were particularly grateful that it *increased family attention to them and their health*: *It’s good for the whole family because it helps us watch our blood pressure” (Male*, *Maori*, *48) and “My grandchildren think it’s a buzz—they like to get involved*.*” (Female*, *Maori*, *57)*.

One story captured family involvement, the value placed on the telecare process and imaginative use of the equipment. As told by one of the staff, this woman had diabetes, poorly controlled for many years.

*One patient came in asking if it was OK*, *she said ’look I trained my husband up to see if he can work the hub* … *because I’ve got to look after the [place where she works] and I don’t want to disconnect anything*. *But what I do is take all my information and I ring him up in the morning [he’s 4 kms down the road from where she’s staying] and I give him all the information and he puts it into the hub for me*. …*oh I think it’s neat*, … *he said*, *that way I can keep an eye on her too* … *for him he’s playing a crucial part*. *It’s the quality of life he’s playing a part of*. *(Primary care clinic staff)*

Clinic staff saw family involvement as a promising advantage of telecare.

…*the clinicians were saying [telecare equipment] would actually assist in not just the individual’s awareness but a whole whānau in terms of just increasing their awareness about health issues and how they might overcome those issues (Primary care manager)*.

For one primary care manager, this was the strongest reason to provide the service.

… *a generation of tomorrow will benefit from the experiences and input that we are going through*.

One primary care clinic staff member felt that telecare had exceeded their expectations for patients by “*making it 10 times better”*.

There were several examples of telecare helping to engage people in their health care when they had been unable to do so previously. One man with diabetes had longstanding difficulty attending health services due to his work as a long-distance truck driver. He established a routine of testing his sugar during work-breaks, recording the readings, and transferring them to the hub when he returned home outside working hours. The nurses would then ring him the following day if needed.

Several patients said that telecare *“saves me going to the doctor*.*” (Male*, *Maori*, *56)*

*The hub takes the weight off the doctors eh*? *Because you are monitoring your own health and once you punch it in the machine it goes walking; it goes somewhere and the results come back instead of being a nuisance to the doctor up there (Patient*, *focus group 1)*.

Similarly, a rural nurse noted:

*I have had less visits about what I am seeing*, *the feedback from the Doco* [Docobo] *that I am getting*, …*I am not having to do weekly visits to say how is it going* (Primary health care rural nurse).

A clinic nurse thought it had increased her profile with patients such that they were confident to approach her rather than a doctor.

*I find it’s been good*. *A lot of the people who would normally come to the doctor two to three times a week* … *they’ll ring me and they’ll say ‘I need to see the nurse because my blood pressure is up a bit’* … *(Primary care clinic staff)*

Some staff questioned why measurements were collected daily and thought that the use of the equipment could have been more customised to each patient. For example, they thought that patients with diabetes may not need their daily measurement for blood pressure and Site C staff decreased this frequency for some patients.

### Technical problems

For several months at the start of the study there were important technical problems with the system. Staff at all sites reported that on multiple occasions data were missing or duplicated. Staff were concerned that their workload increased as they needed to monitor the results electronically, but then contact the patient anyway in cases of missing or duplicate data. There was some loss of trust in the system. Particularly at Site A, this resulted in a change of pattern of clinical care, with the teams checking on the patients more frequently than they otherwise would have, which may have resulted in more outpatient visits or hospital admissions.

After investigating patient use of devices, the devices themselves, connection issues at patients’ homes, and compatibility between devices and the New Zealand telecommunications infrastructure, these problems were eventually sourced to vendor software issues and were finally resolved by early May 2011. In view of these issues we examined time series graphics of the rate of hospital use before and after mid May 2011, and, seeing no pattern of change associated with that date, made no adjustments to the analysis.

## Discussion

The qualitative results suggest that telecare was well received by patients, many of whom felt safer, more knowledgeable about their health, more engaged in self-care and grateful for increased understanding and support from their families. Some staff considered that an increase in family knowledge could be the most important long-term benefit of telecare. Staff were generally positive after initial technical difficulties with the equipment, and reported many instances of improved patient care. However, the quantitative results showed no evidence that telecare improved health related quality of life as measured by the SF36, the main outcome for which this study was powered. Both anxiety and depression scores showed significant, if small, improvements. Point estimates for most other scales moved in the favoured direction. Hospital use and costs did not statistically differ between the groups after adjustments for age, gender, ethnicity, site, and pre-trial hospital use and costs. The results are broadly similar to those from the recent and large Whole Systems Demonstrator randomised controlled trial in the United Kingdom [28–30]. This trial, which also involved people with COPD, CHF and diabetes, found that their version of telecare was not more effective than usual care with respect to quality of life, service use or costs.

Limitations of the current study include a relatively small sample size. Patients randomly allocated to intervention at Site A may have been more unwell or disabled than those in the control group, implied by having more admissions and more days in hospital in the previous year. We believe that the standard of usual care being offered at Site A and Site B was already high, making any effect from telecare smaller and more difficult to detect. The same staff treated both intervention and control groups. In hindsight, this invited contamination between groups, perhaps increasing surveillance of control groups. Including two randomised sites and one intervention-only site, and combining chronic diseases is unusual, but we think we have a sound theoretical basis for doing so, and the sensitivity analyses excluding Site C made little difference to the results. Lack of information about the numbers screened prior to randomisation potentially limits the generalizability of findings. We have noted the technical difficulties with the equipment and data presented for monitoring. Site A staff were more concerned about these difficulties than were the staff at other sites. Although we saw no change in hospital use associated with the date by which the technical difficulties were resolved, such a change would be difficult to detect if any effects on staff behaviour persisted well beyond this date. We are also aware that new technologies are constantly emerging so that, for example, technical issues and costs in this study may not be directly transferrable to alternative systems.

The discordance between the qualitative and quantitative results suggests that large benefits for some patients remained undetected amongst effects averaged over larger numbers of people, and/or that we did not have quantitative measures for the variables that changed. Qualitative enquiry may be more sensitive and subtle with respect to changes that were not anticipated or were experienced by subgroups within a study. Our qualitative data may point us to very specific combinations of context, mechanism and outcomes in which telecare is effective [31]. For example, the long-distance truck driver and the woman who phoned her husband from work so that he could enter the data at home both showed how telecare could reconnect people to healthcare that would otherwise be sacrificed due to the imperatives of employment. Identifying such patients prospectively is likely to need strong theories and an assessment approach that includes symptoms and social circumstances that go well beyond disease labels.

The most striking stories of personal and family engagement came from the rural community (Site C). This community has been traditionally underserved, and the refusal of that community to accept a ‘usual care’ trial arm reflected appropriate advocacy from the local health service providers. Such refusal is common especially from Maori [32] in New Zealand and from minority communities internationally [33]. Rejection of the implications of randomisation also underlay some of the difficulties implementing the large UK Whole Systems Demonstrator project [34].

Site A, in particular, showed an increase in service use amongst the intervention group during the trial compared with before the trail. This result is not uncommon when health services and observation are increased [35]. It may indicate previously unmet need, unrecognised need, changed expectations of service, or perhaps creating more situations that require decisions by health professionals or patients. When more decisions are required, at least some decisions will require review by the clinical team, and when the clinical team is hospital-based this means more admissions, emergency department visits or outpatient visits. We note that Site A has traditionally been secondary care focussed, whereas Site B has a longer institutional history of attempting to support or hand back to primary care. These may all be reasons to connect patients to telecare from primary care rather than secondary care, because increasing patient connection to primary care (with strong secondary care support) is a goal of most health systems, and is likely to be cheaper than increasing patient connection to secondary care. Primary care would seem the appropriate location to implement the holistic assessment approach discussed earlier.

If benefits from telecare are really restricted to specific, poorly defined subgroups of patients, then, at least for the present, future studies may need to eschew the randomised controlled trial, which has been described as an ‘impoverished way to learn’ in a complex environment [34, 36, 37]. Refining theory may be helped by further measuring variables that our qualitative data indicate as important benefits of telecare. These include patient knowledge and health literacy, patient engagement with health services, family engagement with patient healthcare and the potential to shift from doctor to nurse care. Further qualitative assessment might consider the extent to which telecare addressed clinical needs and decisions or whether it more importantly addresses patient (and provider) anxieties and whether this second mechanism leads to altered decisions by patients and professionals with respect to seeking hospital care. Other important questions that remain unanswered are at what stage in a progressive disease process can patients be helped by telecare and how often should each clinical parameter be assessed?

## Supporting Information

S1 CONSORT Checklist (DOC)

S1 Protocol (DOC)
