# Supplementary material for: Telecare for Diabetes, CHF or COPD: Effect on Quality of Life, Hospital Use and Costs. A Randomised Controlled Trial and Qualitative Evaluation
Source: PLoS One. 2015 Mar 13;10(3):e0116188. doi: 10.1371/journal.pone.0116188 (PMC4358961; doi:10.1371/journal.pone.0116188)
Supplement: S1 Protocol — (DOC) [file pone.0116188.s002.doc]

**A.S.S.E.T. Application of Self-management Systems**

**Detailed study protocol**

**4 January 210**

**ASSET Research** Team

| Prof Harry Rea | Principal Investigator | harry.rea@middlemore.co.nz |
| --- | --- | --- |
| Assoc Prof Matthew Parsons | Research leader, Christchurch & Tairawhiti | [m.parsons@auckland.ac.nz](mailto:m.parsons@auckland.ac.nz) |
| Assoc Prof Timothy Kenealy | Research leader, Auckland & Counties | t.kenealy@auckland.ac.nz |
| Assoc Prof Paul Rouse | Finance, cost and resource capture | [p.rouse@auckland.ac.nz](mailto:p.rouse@auckland.ac.nz) |
| Dr Karen Day | Action research, technology implementation | [k.day@auckland.ac.nz](mailto:k.day@auckland.ac.nz) |
| Prof Jim Warren | Technology implementation | [j.warren@auckland.ac.nz](mailto:j.warren@auckland.ac.nz) |
| Dr Koray Atalag | Technology standards and integration | k.atalag@auckland.ac.nz |
| Shereen Moloney | PhD candidate, system changes with telecare | shereen.moloney@auckland.ac.nz |
| Gayl Humphrey | Practical implementation, ADBH | [gaylh@adhb.govt.nz](mailto:gaylh@adhb.govt.nz) |
| Assoc Prof Robert Dougherty | Cardiologist, Auckland | r.doughty@auckland.ac.nz |
| Dr Michelle Honey | Qualitative, consumer views | [m.honey@auckland.ac.nz](mailto:m.honey@auckland.ac.nz) |
| Dr Peter Carswell | Knowledge transfer, quality improvement | [p.carswell@auckland.ac.nz](mailto:p.carswell@auckland.ac.nz) |
| Assoc Prof Nicolette Sheridan | Qualitative, consumer view | n.sheridan@auckland.ac.nz |
| Paul Roseman | ProCare | [paul@procare.co.nz](mailto:paul@procare.co.nz) |
| Dr Patrick McHugh | GP liaison Tairawhiti DHB | [patrickm@tdh.org.nz](mailto:patrickm@tdh.org.nz) |
| Dr Jenni Harre-Hindmarsh | Research manager Ngati Porou Hauora | jennie.harrehindmarsh@nph.org.nz |
| Dr Roland Meyer | Respiratory physician, Christchurch | roland.meyer@canterburyinitiative.org.nz |
| Elizabeth Robinson | biostatistician | e.robinson@auckland.ac.nz |

Contents

[1. Background 4](#__RefHeading___Toc250301980)

[2. Research hypotheses 4](#__RefHeading___Toc250301981)

[3. Study Design 5](#__RefHeading___Toc250301982)

[4. The comparison groups 5](#__RefHeading___Toc250301983)

[5. Ethics 6](#__RefHeading___Toc250301984)

[6. Eligibility 6](#__RefHeading___Toc250301985)

[7. Recruitment 6](#__RefHeading___Toc250301986)

[8. Baseline and repeated assessments 7](#__RefHeading___Toc250301987)

[9. Randomisation and study group assignment 7](#__RefHeading___Toc250301988)

[10. Qualitative interviews 8](#__RefHeading___Toc250301989)

[11. Optimising the Intervention 10](#__RefHeading___Toc250301990)

[12. Describing usual care 11](#__RefHeading___Toc250301991)

[13. Measuring costs 11](#__RefHeading___Toc250301992)

[14. Outcome Measures 12](#__RefHeading___Toc250301993)

[15. Adverse event reporting 13](#__RefHeading___Toc250301994)

[16. Data Handling and Record Keeping 13](#__RefHeading___Toc250301995)

[17. Statistical Issues 15](#__RefHeading___Toc250301996)

[18. Analysis 15](#__RefHeading___Toc250301997)

[19. Timeline 17](#__RefHeading___Toc250301998)

[20. References 18](#__RefHeading___Toc250301999)

[Appendix 1. Baseline data collection 20](#__RefHeading___Toc250302000)

[Appendix 2. Outcome measures 21](#__RefHeading___Toc250302001)

[Appendix 3. Interview schedules 22](#__RefHeading___Toc250302002)

[Appendix 4. Adverse Effect adjudication process and reporting 24](#__RefHeading___Toc250302003)

[Appendix 5. Study flow diagrams Auckland, Counties Manukau and Canterbury 25](#__RefHeading___Toc250302004)

[Appendix 7. Study flow diagram Tairawhiti 26](#__RefHeading___Toc250302005)

[Appendix 8. Workload and equipment needed by month 27](#__RefHeading___Toc250302006)

**List of acronyms and abbreviations**

DHB District Health Board

HRQoL Health related quality of life

DSSI Duke Social Support Index

SF36 Short Form 36

EADL Extended Activities of Daily Living

BOP Bay of Plenty

COPD Chronic Obstructive Pulmonary Disease

AMTS Abbreviated Mental Test Score

# Background

**Telecare** is a new development in New Zealand. It combines **self-monitoring -** whereby people with long term conditions monitor their symptoms to track progress, modify behaviours, or medications accordingly or assess when to seek help from health professionals (Singh, 2005) - with **tele-medicine** which is described as the use of telecommunications technology for medical diagnostics, monitoring, and therapeutic purposes when distance and/or time (or access) separates the participants AHRQ (2006).

**ASSET** is:

1. the first stage of developing a programme of randomised controlled trials to assess the effectiveness of telecare in New Zealand; and
2. a systematic service development that will have multiple outputs of direct value in its own right.

It will be conducted in four study sites (Auckland, Counties Manukau, Tairawhiti and Canterbury). Each study site involves a distinct population group and service model though share some outcomes and telecare product.

**Terminology**

We use the word **‘patient’** to designate the person who is the subject o f care, the person with the long term condition. We use the term **‘family’ or ‘family / whanau’** to designate the others living in the household with the patient, or who provide substantial health care to the patient on the basis of their relationships to the patient, not in a professional relationship. We use the word **‘participant’** to refer to any or all of the patient, family / whanau, and all the health care professionals involved in the patient’s usual care or telecare. We use **‘clinician’ or ‘health professional’** interchangeably to refer to doctors, nurses, pharmacists and other relevant professionals.

It should be noted that the quantitative components of ASSET will develop **pilot data in the New Zealand context to inform sample size calculations** with respect to effect size and frequency of events. **It is not powered to definitively confirm effectiveness for most outcomes** (see power calculations). These requirements will be addressed in subsequent studies.

It should also be noted that ASSET will in effect form a **process map of ‘usual care’**, which is necessary to develop agreement with health care providers on developing and definitively measuring the effects of telecare.

# Research hypotheses

These hypotheses underlie the current ASSET trial *and* the intended follow up trials.

That, compared to ‘usual care’, for people with specific long term conditions, telecare will:

1. Increase the health related quality of life (HRQoL) and satisfaction with health services for patients and family / whänau;
2. Decrease hospital attendance and admissions;
3. Improve the HRQoL of the primary informal caregiver;
4. Provide an economically more efficient means for the person, family / whänau, providers and funding agency to manage a person’s chronic condition;
5. Be feasible and acceptable to patients and health care professionals
6. Be an enabler for the integration of health services;

# Study Design

ASSET will be conducted as:

- **Three separate randomised controlled trials** in Auckland, Counties Manukau and Canterbury (there will be no control group in Tairawhiti)
- Within each of the active intervention arms, including Tairawhiti, there will be an **action research process to optimise the telecare system intervention.**
- Within each control arm there will be a **process of mapping real usual care.**

Each region will stand alone as a discrete trial though with some shared outcomes. Standard criteria and measures will apply across all trials.

The telecare equipment will be available to all four health services for 12 months, with equipment loaned to any one patient for up to three months.

Participants will be assessed at baseline with follow up at three (immediately post intervention) and six months (three months post intervention).

# The comparison groups

The interventions to be evaluated are:

1. the specialist heart failure service with telecare compared with the existing heart failure specialist services in Auckland (for heart failure);
2. the specialist respiratory service with telecare compared with existing respiratory services in Counties Manukau (for COPD);
3. the Ngati Porou Hauora chronic care programme with telecare compared with the existing chronic care management programme in Tairawhiti (for multiple long term conditions); and
4. the community specialist respiratory medicine service with telecare compared with existing respiratory services in Canterbury (for COPD).

# Ethics

We will apply to the Ministry of Health Northern Ethics Committee. No patients will be enrolled before ethics approval is obtained.

A researcher will provide an approved participant information sheet to all people who are potentially eligible to participate. In Auckland, Counties Manukau and Canterbury, those who provide written consent will have baseline data collected and will then be randomly allocated to intervention or control arms. From those who do not consent to participate we will seek to obtain basic demographic information. In Tairawhiti all participants will be included in the active development and observation programme.

# Eligibility

**Inclusion criteria:**

1. *All areas*
   1. age 16+
   2. living permanently at home
   3. expected to be available for 3 months
   4. expected to survive study period
   5. able to communicate in English (or someone in household able to translate)
   6. able to physically manage the equipment (or someone available to assist)
2. A*uckland* – Permanently resides in the Auckland DHB region, current or recent in-patient to Auckland City Hospital with heart failure as the primary reason for admission
3. *Counties Manukau* – Permanently resides in the Counties Manukau DHB region, enrolled with ProCare PHO, current or recent in-patient at Middlemore Hospital with COPD as the primary reason for admission
4. *Tairawhiti* – Permanently resides in the Tolaga Bay area, enrolled with Ngati Porou Hauora PHO, and is on the PHO chronic condition management programme
5. *Canterbury* – Permanently resides in the Canterbury DHB region, enrolled with practices working with the community specialist respiratory service, with COPD being treated with Long Term Oxygen Therapy (LTOT) at home

**Exclusion criteria:**

1. Significant cognitive impairment (<8/10 Abbreviated Mental Test Score - AMTS)
2. Serious current physical or mental illness
3. Previously used telecare

# Recruitment

**Numbers potentially available**

*Auckland* – about 5000 known patients with CHF, about 500 admissions, about 300 admissions with ‘new’ CHF per year

*Counties Manukau* - About 600 ‘new’ COPD patients are admitted to hospital each year

*Canterbury* - about 500 ‘new’ COPD patients are admitted to hospital each year; 250 on LTOT at any one time

*Tairawhiti* – to a maximum of 50 participants at any one time

**Recruitment strategies:**

*Auckland* – identified by CNS on admission with CHF, screened for eligibility and consent to pass name to researcher, who will conduct formal consent process.

Counties Manukau - identified by CNS on admission with COPD, screened for eligibility and consent to pass name to researcher, who will conduct formal consent process.

Tairawhiti – identified by chronic care nurse, from current register of community patients, she will screen for eligibility and conduct formal consent process.

Canterbury - identified by CNS on admission with COPD, screened for eligibility and consent to pass name to researcher, who will conduct formal consent process.

# Baseline and repeated assessments

All study participants, in intervention and control groups, will have the same data collected relating to quality of life and resource use at baseline, 3 months and 6 months.

Those in the intervention arm will be asked additional questions relating to the technology, at 3 months and 6 months.

For baseline data collection see **Appendix 1**.

# Randomisation and study group assignment

Immediately after baseline data collection, participants in Auckland and Counties Manukau will be randomised to intervention or control arms. The researcher will phone an Interactive Voice Response (IVR) system via an 0800 number, provide basic patient identification data and be assigned a study group for that patient.**

*Eligible 🡪 consent 🡪 baseline data collection 🡪 individual randomly assigned to intervention or control*

In Christchurch, a pre-assigned cluster randomisation will assign participating general practice surgeries – and the patients attending them – to intervention or control arms of the study.

*Eligible 🡪 consent 🡪 baseline data collection 🡪 cluster randomised to intervention or control, based on randomly assigned group of patient’s general practice*

In Tairawhiti, patients will not be assigned to separate groups.

*Eligible 🡪 consent 🡪 baseline data collection 🡪 followed in telecare programme*

Note: initially we intended to randomise with stratification by age / gender / distance from hospital / ethnicity. We have decided against this as i) we are collecting pilot data and will find if we obtain an adequate stratification by including all who are eligible and consent ii) there are technical and efficiency reasons not to stratify. From this pilot data we will be better informed as to whether stratification will be necessary in a follow up trial.

# Qualitative interviews

While the core research in this project is aimed at establishing the purpose, design and evaluation of telemonitoring as a clinical intervention, there are other key components of telehealth that lend themselves to measurement. They include

- **Business implications of telemonitoring**
  - How ‘usual care’ is performed as a business process in both primary and secondary care (processes as they are currently done/used)
  - What the implications of telemonitoring are on ‘usual care,’ e.g. what happens when care is shared between specialist, primary care doctor and chronic care nurse, and how this contributes to the concepts of ‘integrated care’ or ‘shared care’ (process changes as a consequence of telemonitoring)
- **Chronic condition management implications**
  - What the impact is on chronic condition self management, and how the potential for self care can be supported using telemonitoring (self care as it is currently done and consequent changes resulting from telemonitoring)
- **Criteria for including people in a telemonitoring mode of** **care.** It is not clear in the literature how people are selected for telemonitoring. Most of the literature surveyed provides evidence THAT there are benefits associated with telemonitoring, but no clear guidance is given for deciding how to select users.
- **Criteria for exiting people from a telemonitoring mode of care.** It is not clear how a decision is made that a person either no longer benefits from telemonitoring or would benefit more from exiting such a programme.
- **Cost analyses of using telemonitoring.** The literature does not provide complete, sufficient or comprehensive information on cost analyses.

Potential participants for this set of research projects include the ‘actors’ listed below as described by Mantzana et al ^1^ (in no particular order). These participants will be identified using the Balanced Score Card and the person centric continuity of care model for people with chronic conditions developed by Warren et al ^2^.

- **In primary and secondary care services,** clinicians (doctors, nurses and allied health care personnel associated with people receiving care for chronic conditions), managers and administrative staff will be approached to participate in this research to
  - **Before recruiting patients for telemonitoring**
    - Describe current care processes and identify clinical guidelines and protocols in use as benchmark of best practice for people with chronic conditions relating to this project ^3 4^
    - Reflect on and document current ‘usual care’ and associated processes, workflow and routines e.g. emergency care admission process of patients already receiving specialist care ^5^.
  - **After one year of patients using telemonitoring,** in a semistructured interview, they will be asked the questions outlined in Appendix 3.
- **In the community,**  people with chronic conditions (as identified by their clinicians for inclusion in the telemonitoring project), their family members (and Whanau) and their extended social supports (friends, co-workers and others deemed appropriate for participating in the research) will be approached as follows.
  - **‘Patients’ will be invited to participate regarding their perceptions of self management of their chronic condition as follows**
    - The ‘patients’ will be interviewed regarding their self-care capacity, using the questionnaires from the Flinders model ^6^. The twelve questions from the Partners in Health Scale (see Appendix 3) will be used to establish the degree to which patients are managing their own condition.
    - Further questions will be asked in terms of patient perceptions of their clinical outcome from the telemonitoring intervention and compared with that of their clinicians.
    - They will be asked these questions at the beginning and end of their use of telemonitoring to establish if the intervention made any change to their sense of self management.
  - **‘Patients’ and their family members who share care with them will be asked about their propensity to use technology as follows.**
    - Based on the Technology Adoption Model by Davis as used by Rahimapour et al ^7^ we will ask questions about perceptions of usefulness of the telemonitoring kit (see Figure 2 for the questions). These questions will be used as a guide to frame reflection about how the technology was seen to be useful for self management. Other questions are added as indicated in Appendix 3.
    - This semistructured interview will be done in the third month in which a ‘patient’ uses the telemonitoring kit.

**The ASSET project team will be invited to reflect** on the following questions

- How does the telehealth intervention change equity, equality, empowerment, trust among clinicians, monitoring of patients in their home, effect of surveillance, therapeutic or dehumanizing effect, different meanings of information? ^5^
- To what extent does the information system meet the project’s requirements as well as objectives?^3^
- What can be generalised/transferred to another study/setting?^5^

In addition, the team members will be asked to reflect on the project using the REFLECT mnemonic as outlined by Day, Orr, Sankaran & Norris ^8^ as follows:

“...**R**eview thoughts, feelings, behaviour linked to planned and unplanned action; we look for **E**xceptions when everything appears to be going well; we consider the **F**uture impact such as identifying the next step in our activities; we **L**earn as we consider opportunities for building models and enhancing our understanding; we seek **E**xplanations when things are in apparent disagreement; and we **C**hallenge assumptions and consider what **T**roubles or puzzles us about our observations.” (p. 3).

This form of reflection will be conducted at the end of the first year and will be used to float ideas, thoughts and observations about the intervention that could be useful for future research. Using a complexity theory approach ^9^, this form of reflection can raise emerging concepts, patterns and practices in ways that can be further researched.

# Optimising the Intervention

The chosen approach is action research (AR). For those not familiar with this method, it is a cyclical process of plan, act, reflect and learn, and adapt the planned activities to enhance the final outcome of what we endeavour to achieve ^10^. It is broader than a ‘plan do study act’ quality improvement cycle. Its mixed heritage includes anthropological campaigns that sought to challenge social structures by using research understanding to empower the least powerful in society. More pragmatically, the processes have proven useful to iteratively clarify the requirements and concerns of those involved in deploying or using new technologies.

Action research implies cycles and iterations of learning and application ^10 11^. For the ASSET project action research will be used as follows:

- The current project defined as the exploration of telemonitoring in New Zealand, and as formative pilot study for future randomised clinical trials on telemonitoring, is the primary and encompassing AR cycle, and
- Each three month allocation of telemonitoring for a patient will be considered a cycle of planning, acting and learning.

AR also implies that a problem has been identified, is being clarified and resolved ^10^. For three DHBs the clinicians and healthcare service providers have identified a chronic condition management issue that telemonitoring could contribute to with positive outcomes, while Tairawhiti DHB has a different approach. The Tairawhiti community will be invited to participate with clinicians and service providers to identify the problem/s for which telemonitoring could contribute a solution.

More specifically, the researchers will:

- observe those using the technologies, primarily the clinical nurse specialists, patients / whanau, each for about 4 periods of one hour over 3 months
- conduct semi-structured interviews with each at the beginning and end of the intervention
- iteratively review their findings with each party at the end of each observation period
- record changes they observe as the intervention is refined
- seek to establish and document consensus on an algorithm of care the CNS will use for a further trial

# Describing usual care

The researchers will use an observation and interviews, as above, to decribe and document ‘usual care’ at each site.

As part of this process we will ask each CNS to keep a diary of costs and resource use for 1 month, and each patient/whanau participants to keep a diary of costs and resource use for 2 weeks.

In addition, each patient in the usual care group will be asked to complete the same interview and questionnaire schedule as the Intervention group, less the technology-specific components.

# Measuring costs

The resources consumed and services utilised by patients will be measured for both groups with particular effort to identify the resources being used by the intervention group. The analysis will take a societal perspective and include costs from the health sector, other government services and the informal sector as much as possible. Costs will be adjusted to represent a single financial year. For each client, information will be required on number and length of hospitalisations, falls and number of contacts with case managers. Where direct costs are not available for individual patients (from the Patient Cost System) average costs per unit of service provided will be used.

Assuming that the concern is about discount priced technology for the trial, this will affect depreciation and the investment cost. My feeling is that we can do some sensitivity analysis that incorporates 'actual' prices (if these are discounted) and 'realistic' prices (if we feel the discounted prices are abnormal). One has to bear in mind that prices for technology tend to decline fairly rapidly so that even if the trial product prices are low, it is likely that they will be sustainable because of future declines in pricing. However, a sensitivity analysis will show how material any price differences will be on the economic evaluation

The following items will be determined as follows:

Intervention – total outlay on technical equipment and one-off expenditure

Costs pertaining to the intervention group eg depreciateion, monitoring, communications, home visits; costs pertaining to tht control group eg home visits / nurse time.

Benefits – difference in costs of hospitaliseation before and after and between groups.

Recording via chromosomes

Daily log

- What are the investment, operational costs, cost benefits, and return on investment?^3^
- Cost analysis^4^
- Time (to complete the process &/or activities associated with the technology) ^4^

# Outcome Measures

*Primary Scales*

1. HRQoL measured using the 36-item Short Form (SF-36) questionnaire

2. Self-efficacy measured on scale Self-Efficacy for Managing Chronic Disease 6-item scale

3. Disease-specific scales

a. COPD - St George Respiratory Questionnaire^12^; BODE Index^13^; COPD Helplessness Scale^14^

b. CHF – Minnesota Living with Heart Failure Questionnaire^15^

c. Other conditions -

*Primary quantitative health outcomes and resource use*

Hospital ED attendance, admissions, length of stay - all cause and disease-specific

*Secondary* – physical function, number and length of admissions to acute hospitals as well as time between initial and subsequent admission, number of GP consultations, quantity and quality of social support networks, and adverse events (falls and other injuries) and sense of control in chronic care consumer; HRQoL and mental wellbeing in carer or family (whänau); adoption of self management programme; adoption of telecare equipment and associated processes and workflow changes by participants, including organisational impact, e.g. changes in ASH rates, satisfaction with services.

*Financial* – Direct and indirect economic costs in the consumer, caregiver or family (whänau), and health service provider. Cost data will be collected for both intervention and control group costs for attending hospital. Technology costs for the intervention group only. Financial benefits will be calculated by the difference in admission and treatment costs between the two groups. Costs will be calculated using the ongoing cost of monitoring and interventions. The net cost savings or benefit will allow the return on investment to be calculated.

# Adverse event reporting

All of the people enrolled in this study are under ‘usual care’ – from their general practitioner and / or hospital specialists - with or without the addition of telecare. Given that the participants are either unwell or may become unwell, possibly at short notice, we must allow for the possibility that introducing telecare may disrupt care in ways not anticipated.

Adverse events to be recorded and assessed by independent review, for intervention group only, within 6 months of enrolling in study:

1. Death
2. Unplanned hospitalisation or prolongation of hospitalisation
3. Patient withdraws from study

A **Data and Safety Monitoring Board Panel** (DSMP) will be appointed to undertake and / or oversee these reviews. The Panel will include clinical, research and statistical skills. Analyses will be un-blinded.

See **Appendix 2** for adjudication process and reporting

# Data Handling and Record Keeping

****Sarah**

**Confidentiality**

Information about study participants will be kept confidential and managed according to the requirements of The Code of Health and Disability Services Consumers' Rights, 1996, and the Ethics approval specific to this study.

**Source Documents**

Source data is all information, original records of clinical findings, observations, or other activities in a clinical trial necessary for the reconstruction and evaluation of the trial. Source data are contained in source documents. Examples of these original documents and data records include: hospital records, clinical and office charts, completed interview schedules, and subject files involved in the clinical trial.

**Case Report Forms**

The study case report form (CRF) is the primary data collection instrument for the study. All data requested on the CRF must be recorded. All missing data must be explained. If a space on the CRF is left blank because the procedure was not done or the question was not asked, write “N/D”. If the item is not applicable to the individual case, write “N/A”. All entries should be printed legibly in black ink. If any entry error has been made, to correct such an error, draw a single straight line through the incorrect entry and enter the correct data above it. All such changes must be initialled and dated. **Do not erase or white-out errors.** For clarification of illegible or uncertain entries, print the clarification above the item, then initial and date it.

**Records Retention**

It is the local researcher’s responsibility to retain study essential documents and deliver these to the Study Manager at the cessation of the study. The Study Manager will ensure these records are retained securely for a period of 10 years from collection, or otherwise as indicated in the Ethnics approval specific to this study.

**Data and document handling**

The following table outlines the flow of information from recruitment to assessment.

Table 1: summary of data and document handling

| **Record** | **Data handling procedure** |  |
| --- | --- | --- |
| Eligible clients list | Stored electronically by local researcher; emailed to Study Manager weekly |  |
| Ineligible clients list | Stored electronically by local researcher; emailed to Study Manager weekly |  |
| Contact list | Stored electronically by local researcher; emailed to Study Manager weekly |  |
| Declined clients list | Stored electronically by local researcher; emailed to Study Manager weekly |  |
| Delayed Interview list | Stored electronically by local researcher; emailed to Study Manager weekly |  |
| Randomisation list | Auckland and Counties Manukau: researcher phones randomization service while still with patient, records on Care Report Form, updates local list of Intervention and Control patients, emails Study Manager within 24 hours.  Canterbury: researcher confirms general practice of patient, confirms and records Intervention or Control group, emails Study Manager within 24 hours. |  |
| Enrolled participant list | Local researcher maintains up-to-date list; emails to Study Manager weekly |  |
| Participant contact card | Local researcher maintains up-to-date list; emails to Study Manager weekly |  |
| Withdrawn participant list | Local researcher maintains up-to-date list; emails to Study Manager weekly |  |
| Participant research interview (CRF = Case Report Form) | Local researcher keeps original, faxes or emails Study Manager weekly. Transfers originals to Study Manager at end of study. |  |
| Interview recordings | Digital recording and field notes sent to Study Manager within 24 hours. |  |

# Statistical Issues

**Potential patients available and recruitment rate**

We estimate that about two thirds of those invited to participate will consent, about 20% will drop out. In addition, for COPD and heart failure, we estimate that the death rate will be about 20% per year.

**Sample size**

Each region will be a stand-alone and discrete trial though with some shared outcomes. Standard criteria and measures will apply across all trials. A sample size of 120 within Auckland, Counties Manukau and Canterbury will provide a power of 90% with alpha = 0.05 to detect a 10 point increase in SF36. Similarly, the literature (Bourbeau) suggests we need 86 patients in each study arm to have 90% power (alpha 0.05) to detect a mean change of 4 (the minimum important difference) on the St George Respiratory Questionnaire. Detecting some other outcomes will require more patients.

See separate document, “ASSET planning and power calculations” for details of data underlying power calculations.

# Analysis

Primary quantitative analysis will be by Intention to Treat (i.e. as planned) and modified Intention to Treat (i.e. as delivered). Exploratory analysis will assess the influence and associations of explanatory and potentially confounding variables. The findings will help determine whether randomization for subsequent trials needs to be simple or stratified.

Subgroup analysis will be employed, where possible with the number of patients in each group, to detect clinically important differences between subgroups that may be defined by age, gender, ethnicity, rural-urban dwelling and severity of initial health assessments.

There will be no imputation of missing data. All analyses will be conducted in Stata. Continuous variables will be analysed using mixed effects models that included site as a fixed effect, patient as a random effect and adjusted for age and gender. The main outcome for the psychometric scales will be assessed from the interaction between intervention group and time. Negative binomial regressions will be used to model count data including numbers of hospital attendance, length of stay and costs in analyses were adjusted for patient age, gender, ethnicity, and site as a factor variable. Exposure, for hospital use, will be measured by days alive and not in hospital. Exposure, for total days in hospital, will be measured by days alive. Results will be considered statistically significant when p ≤ 0.05.

**Qualitative analysis**

Qualitative analysis will be principally use thematic analysis, where the themes are derived from the purposes of the project; themes are implied in most of the questions in the qualitative interview schedules, as the questions themselves are mostly the results of others’ qualitative analyses.

**Cost-effectiveness telecare and usual care**

Analyses will be performed to ascertain the robustness of any cost-utility differences found between the programmes based on this costing procedure. This will include the following tests:

1. Varying the projected technology costs to determine sensitivity to return on investment
2. Varying the percentage of costs assumed for staff costs and related overheads
3. Total versus marginal costs for the different programmes
4. Varying the costs attributed to informal care

**Cost utility analysis**

Model of how much time looking after telecare pt should take v how long it took to actually do and resources involved? What was taking us 3 hours now takes us 2. Ie modelling after learning has taken place and reached a ‘steady state’.

# Summary of data collection at each time point

|  | **At beginning of each patient telemonitoring** | **At end of each patient telemonitoring** | **At end of project** |
| --- | --- | --- | --- |
| **patients** | **Baseline data collection**  **SF36**  **Stanford self-efficacy scale**  **Semistructured interview based on PIH**  **Disease specific questionnaire**  **1. St George Respiratory Questionnaire**  **2. COPD Helplessness Index**  **3. Minnesota Heart Failure questionnaire (?)** | **SF36**  **Stanford self-efficacy scale**  **Semistructured interview based on PIH**  **Disease specific questionnaire**  **1. St George Respiratory Questionnaire**  **2. COPD Helplessness Index**  **3. Minnesota Heart Failure questionnaire (?)** |  |
| **Patient whanau / support people** | **Semistructured interview based on PIH** | **Semistructured interview based on PIH** |  |
| **clinicians** |  |  |  |
| **Clinicians, administrators** |  |  |  |
| **Research team** |  |  | **Interview using REFLECT model** |

# Timeline

| ID | Name | Start | Finish |
| --- | --- | --- | --- |
| 1 | Planning and ethics |  |  |
|  | Detailed protocol | Nov 09 |  |
| 4 | Development of interviews and questionnaires | Dec 09 |  |
|  | Maori consultation | Jan 10 |  |
| 2 | Ethics application | Jan 10 |  |
|  | Vendor contracts signed | Feb 10 |  |
|  | Delivery of first equipment | Mar 10 |  |
| 3 | Recruitment of staff | Feb 10 |  |
|  | Testing of equipment | Mar 10 |  |
|  | Training for equipment | Mar 10 |  |
|  | Training for research protocol, interviewing, questionnaire admin | Mar 10 |  |
| 5 | Data collection |  |  |
|  | First rollout | May 10 |  |
|  | Second rollout | Jun 10 |  |
|  | Third rollout | Jul 10 |  |
| 17 | Analysis and reports |  |  |
| 18 | Data analysis |  |  |
| 20 | Draft report |  |  |
| 21 | Final report |  |  |

# References

# Appendix 1. Baseline data collection

**Patients and family / whanau**

Date of birth, age, Gender

Ethnicity – self-assigned using standard census question

Address, Contact details

Marital status, Household members

Household member / Caregiver details, contacts details

Highest education

Employment status, current and past main employment

Income or benefit status

Social support (eg home help)

Health care support (including GP)

Index condition

Other morbidities

Random assignment group

# Appendix 2. Outcome measures

|  | Primary quantitative |  |
| --- | --- | --- |
| 1 | Number of hospital admissions in 1 year | DHB database |
|  | Number ED attendances | DHB database |
| 2 | Number of bed days in 1 year | DHB database |
| 3 | Deaths | DHB database |

|  | Secondary quantitative |  |
| --- | --- | --- |
| 4 | Change in hospital admissions from previous year | DHB database |

|  | Qualitative & descriptive |  |
| --- | --- | --- |
| 5 | Social problems identified and addressed | See interview schedules |
|  | Organisational processes | See interview schedules |
| 13 | Patient experiences | SF36, disease specific scales, interviews |
| 14 | Whanau / carers experiences | Interviews |
| 15 | Staff (include safety concerns) | Interviews, action research process and observations |

|  | Quality of Care |  |
| --- | --- | --- |
| 7 | Fidelity of care to intended model | Action research, interviews with staff |
| 8 | Measures of integration | Interviews with staff and researchers |
|  | Disease specific measures | To be advised by disease experts researchers |

|  | Costs, Resources |  |
| --- | --- | --- |
| 9 | Standard cost per admission | DHB accounting |
| 10 | Standard cost per bed day | DHB accounting, Patient Cost System |
| 11 | FTE staff on project | DHB management, log by researchers & staff |
| 12 | Additional costs - transport | Staff log |
| 16 | Staff Interventions | Staff diary |
|  | Costs of usual care | Log by staff |
|  | number of people / agencies visiting house | Researcher, patient interviews |
|  | Number of GP visits | Researcher, patients interviews |

# Appendix 3. Interview schedules

**Interviews with patients, about self-care, at the beginning and end of their telemonitoring**

Semi-structured interviews with patients, based on 12 areas of the Flinders Partners in Health Scale.^6^

1. Knowledge of condition
2. Knowledge of treatment
3. Ability to take medication
4. Ability to share in decisions
5. Ability to arrange and attend appointments
6. Understanding of monitoring and recording
7. Ability to monitor and record
8. Understanding of symptom management
9. Ability to manage symptoms
10. Ability to manage the physical impact
11. Ability to manage the social and emotional impact
12. Progress towards a healthy lifestyle.

**Interviews with patients, about their perceptions of the technology, at the end of their telemonitoring**

Semi-structured interview to establish perceptions about usefulness of the technology (based on protocol by Rahimapour et al ^7^, and added to by considerations suggested by Kaplan & Shaw ^5^ and van der Loo et al ^4^).

Participants will be asked about

- Their overall attitude toward the telemonitoring technology – do they like it and what do they like?
- Perceptions about how easy/difficult it is to use the technology, and what they feel are advantages/disadvantages to using it. What their preferences are regarding using the technology.
- Their confidence in using the technology and any possible barriers to building confidence. Any abilities they consider essential for using the technology? What would make them not be able to use the technology?
- How they perceive the technology could help them manage their health.
- How they feel the technology would help them gain more knowledge about their health/chronic condition.
- Perceptions about their clinicians’ use of the technology and the resulting information about them.
- Sense of distance of clinicians now that they are not cared for in hospital. How do they feel about the reduction in face-to-face communication with their clinicians?
- Concerns about privacy and/or confidentiality.
- Their training experience and how they felt it could be improved.
- Their perception on how this technology may changedor has changed their hospitalisations and/or use of emergency care centre.
- Sense of impact on costs to themselves and/or the healthcare services they use.
- Perception of impact of the technology on their time – how does the technology change the way they use their time for self care and/or care delivered by others to them?
- Sense of how the technology impacts on their family, Whanau, friendships and work relationships (for those who are employed).
- Unintended consequences, unexpected experiences, e.g. performance of the technology.
- Perception of satisfaction with the technology and associated care processes.
- Knowing what they know now, why would they use telemonitoring again if they got an opportunity?

**Interviews with health care providers, perceptions of the projected, after one year.**

Semi-structured interview schedule for clinicians, managers and administrators associated with the telemonitoring project, and will cover the changes, assumptions, expectations, outcomes and unintended consequences of the project. Questions based on findings of Kaplan & Shaw ^5^, Ammenwerth et al ^3^, van der Loo et al ^4^ and Callen & Westbrook ^16^.

- Reflect on and document their understanding of the implications of the consequences of telemonitoring on ‘usual care’ processes
- Create guidelines for inclusion of patients in telemonitoring and exit criteria for terminating telemonitoring
- Reflect on the project management implications, requirements and critical success factors for implementing telemonitoring, and identifying the requirements for upscaling telemonitoring as a national initiative.
- Indicate if their sense of job satisfaction has been influenced by the use of telemonitoring
- Reflect on perceptions of usefulness of the technology and resulting information flows, and whether the technology delivered what was expected.
- Consider if/how the use of the technology changed/improved the quality of care for their patients
- The role of policy and culture on the intention to use telemonitoring as a chronic condition management intervention, and how these need to change to ensure further adoption
- What problem areas are there regarding daily use of the technology? Any pitfalls, and solutions?
- Is the technology usable in the intended environment and is it used as intended?
- Why did the outcomes (clinical, process, organisational, protocols) happen the way they did?
- Unintended consequences, unexpected experiences, e.g. performance of the technology.
- Knowing what you know now (after a year), why would you continue to use telemonitoring? What would they improve/change?

**Interviews with ASSET research team, reflections on project, after one year**

- How does the telehealth intervention change equity, equality, empowerment, trust among clinicians, monitoring of patients in their home, effect of surveillance, therapeutic or dehumanizing effect, different meanings of information? ^5^
- To what extent does the information system meet the project’s requirements as well as objectives?^3^
- What can be generalised/transferred to another study/setting?^5^
- **R**eview thoughts, feelings, behaviour linked to planned and unplanned action
- **E**xceptions when everything appears to be going well
- **F**uture impact such as identifying the next step in our activities
- **L**earn as we consider opportunities for building models and enhancing our understanding
- **E**xplanations when things are in apparent disagreement
- **C**hallenge assumptions and consider
- what **T**roubles or puzzles us about our observations (Day, Orr, Sankaran & Norris ^8^)

# Appendix 4. Adverse Effect adjudication process and reporting

Hospitalization, Prolonged Hospitalization or Surgery

Neither the condition, hospitalization, prolonged hospitalization, nor surgery are reported as an adverse event if they are for diagnostic or elective surgical procedures for a pre-existing condition. Surgery should ***not*** be reported as an outcome of an adverse event if the purpose of the surgery was elective or diagnostic and the outcome was uneventful.

Recording

At each contact with the subject, the researcher must seek information on adverse events by specific questioning. Information on all adverse events should be recorded immediately in the source document, and also in the appropriate adverse event section of the case report form (CRF). Where an adverse event occurs between interviews, the Study Manager coordinators will provide the local researcher with the adverse event information.

Report to Study Manager within 1 month

At the time of the initial report, the following information should be provided:

| - Subject number - A description of the event - Date of onset - Current status - The reason why the event is classified as serious - Researcher assessment of the association between the event and study participation |
| --- |

Within the following 48 hours, the researcher must provide further information on the serious adverse event in the form of a written narrative. This should include a copy of the completed Serious Adverse Event form, and any other diagnostic information that will assist the understanding of the event.

Participant Withdrawal

A withdrawal is defined as a withdrawal of consent to participate post-randomisation. The researcher will request the opportunity to ask the client or next of kin some questions after four months and then after 12 months to ascertain if they are alive and where they are residing. The older person or next of kin will have right of refusal.

Medical Monitoring

safety monitoring will include careful assessment and appropriate reporting of adverse events as noted above, as well as the construction and implementation of a site data and safety-monitoring plan. Medical monitoring will include a regular assessment of the number and type of serious adverse events.

# Appendix 5. Study flow diagrams Auckland, Counties Manukau and Canterbury


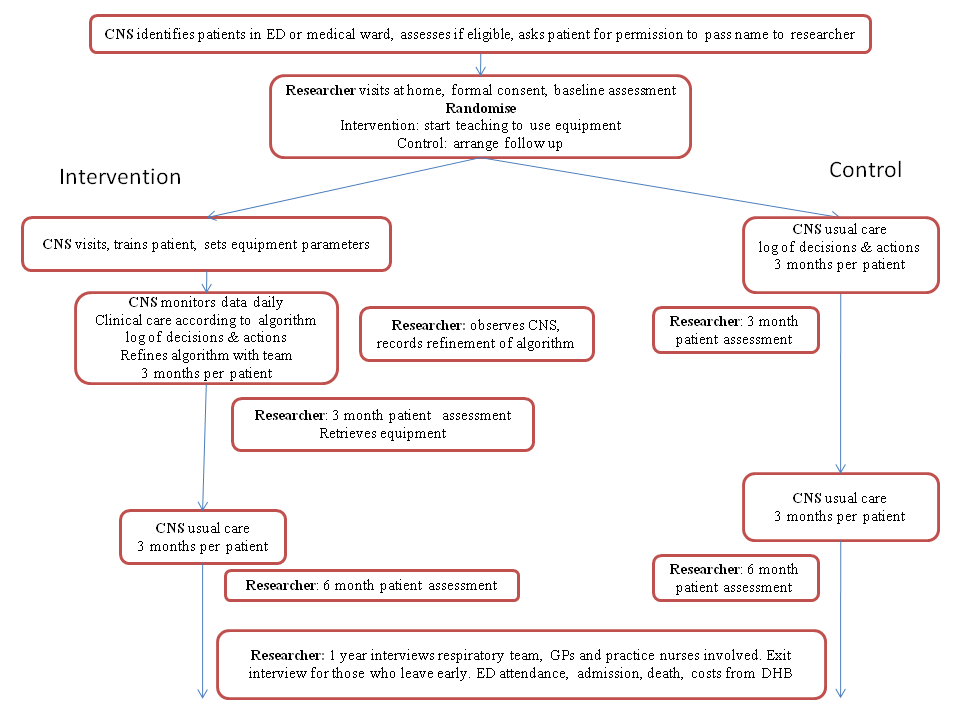


# Appendix 7. Study flow diagram Tairawhiti


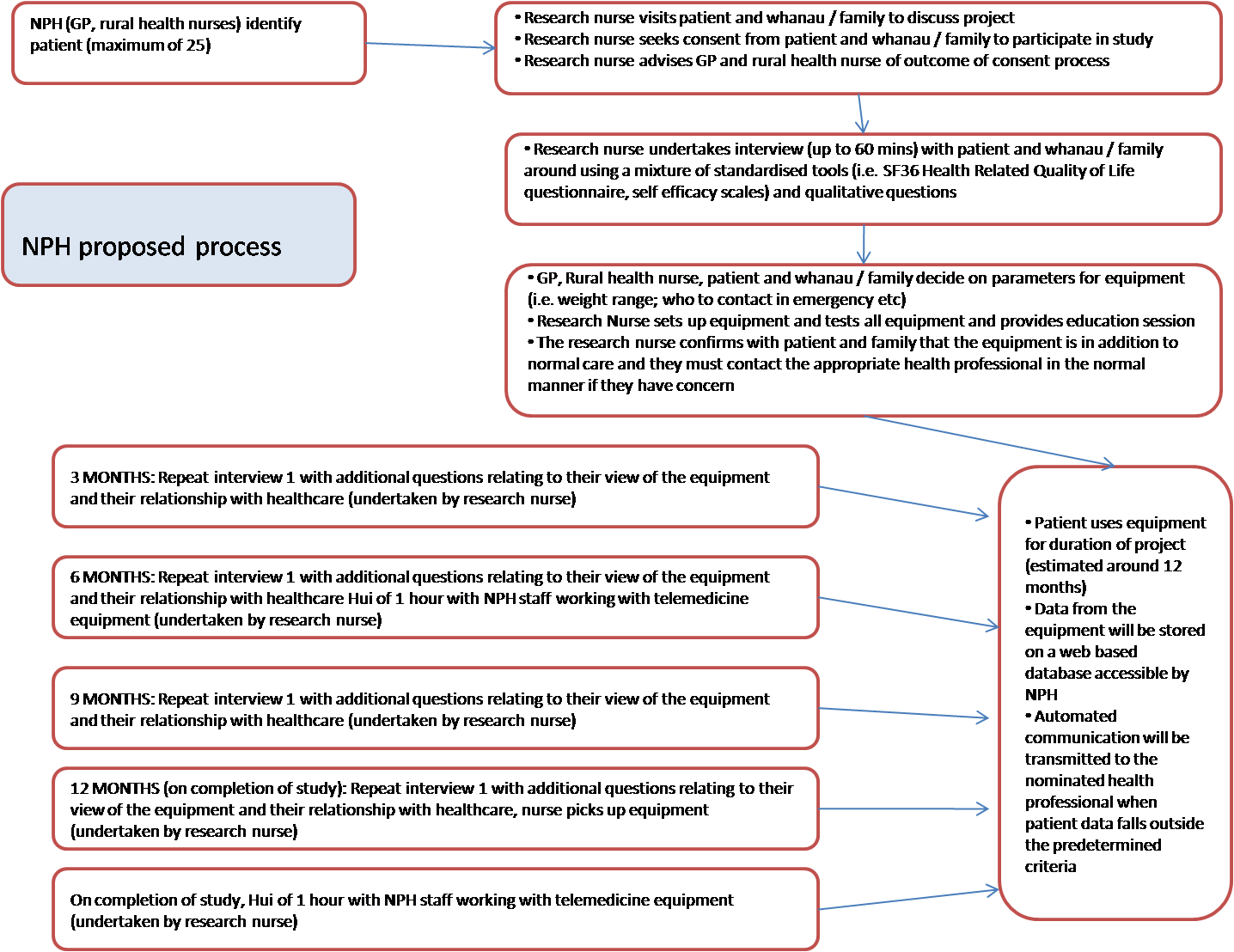


Note detail refinements from J H-H email 24 Nov

# Appendix 8. Workload and equipment needed by month

| **Auckland** |  |  |  |  |  |  |  |  |  |  |  |  |  |  |  |  |  |  |
| --- | --- | --- | --- | --- | --- | --- | --- | --- | --- | --- | --- | --- | --- | --- | --- | --- | --- | --- |
|  |  |  |  |  |  |  |  |  |  |  |  |  |  |  |  |  |  |  |
| Patient numbers in active and control arm, with tallies for clinical load, equipment numbers, researcher load | | | | | | | | |  |  |  |  |  |  |  |  |  |  |
| **equipment used 4 times, 3 months per patient** | | |  |  |  |  |  |  |  |  |  |  |  |  |  |  |  |  |
| recycle equipment starts Jun 10 | |  |  |  |  |  |  |  |  |  |  |  |  |  |  |  |  |  |
|  |  |  |  |  |  |  |  |  |  |  |  |  |  |  |  |  |  |  |
|  | **Mar-10** | **Apr-10** | **May-10** | **Jun-10** | **Jul-10** | **Aug-10** | **Sep-10** | **Oct-10** | **Nov-10** | **Dec-10** | **Jan-11** | **Feb-11** | **Mar-11** | **Apr-11** | **May-11** | **Jun-11** | **Jul-11** | **Aug-11** |
| **Clinical load** |  |  |  |  |  |  |  |  |  |  |  |  |  |  |  |  |  |  |
| recruitment total | 8 | 8 | 8 | 8 | 8 | 8 | 8 | 8 | 8 | 8 | 8 | 8 |  |  |  |  |  |  |
| recruitment to intervention | 4 | 4 | 4 | 4 | 4 | 4 | 4 | 4 | 4 | 4 | 4 | 4 |  |  |  |  |  |  |
| intervention total current | 4 | 8 | 12 | 12 | 12 | 12 | 12 | 12 | 12 | 12 | 12 | 12 | 8 | 4 |  |  |  |  |
|  |  |  |  |  |  |  |  |  |  |  |  |  |  |  |  |  |  |  |
| **Equipment load** |  |  |  |  |  |  |  |  |  |  |  |  |  |  |  |  |  |  |
| equipment in use | 4 | 8 | 12 | 12 | 12 | 12 | 12 | 12 | 12 | 12 | 12 | 12 | 8 | 4 |  |  |  |  |
|  |  |  |  |  |  |  |  |  |  |  |  |  |  |  |  |  |  |  |
| **Researcher load** |  |  |  |  |  |  |  |  |  |  |  |  |  |  |  |  |  |  |
| new patient training | 4 | 4 | 4 | 4 | 4 | 4 | 4 | 4 | 4 | 4 | 4 | 4 |  |  |  |  |  |  |
| patient baseline assessment | 8 | 8 | 8 | 8 | 8 | 8 | 8 | 8 | 8 | 8 | 8 | 8 |  |  |  |  |  |  |
| patient 3 month assessment |  |  |  | 8 | 8 | 8 | 8 | 8 | 8 | 8 | 8 | 8 | 8 | 8 | 8 |  |  |  |
| patient 6 month assessment |  |  |  |  |  |  | 8 | 8 | 8 | 8 | 8 | 8 | 8 | 8 | 8 | 8 | 8 | 8 |
| total assessments + training | 12 | 12 | 12 | 20 | 20 | 20 | 28 | 28 | 28 | 28 | 28 | 28 | 16 | 16 | 16 | 8 | 8 | 8 |
|  |  |  |  |  |  |  |  |  |  |  |  |  |  |  |  |  |  |  |
| **Total patients experiencing telecare system** | | | **48** |  |  |  |  |  |  |  |  |  |  |  |  |  |  |  |

| **Counties** |  |  |  |  |  |  |  |  |  |  |  |  |  |  |  |  |  |  |
| --- | --- | --- | --- | --- | --- | --- | --- | --- | --- | --- | --- | --- | --- | --- | --- | --- | --- | --- |
|  |  |  |  |  |  |  |  |  |  |  |  |  |  |  |  |  |  |  |
| Patient numbers in active and control arm, with tallies for clinical load, equipment numbers, researcher load | | | | | | | | |  |  |  |  |  |  |  |  |  |  |
| **equipment used 4 times, 3 months per patient** | | |  |  |  |  |  |  |  |  |  |  |  |  |  |  |  |  |
| recycle equipment starts Jun 10 | |  |  |  |  |  |  |  |  |  |  |  |  |  |  |  |  |  |
|  |  |  |  |  |  |  |  |  |  |  |  |  |  |  |  |  |  |  |
|  | Mar-10 | Apr-10 | May-10 | Jun-10 | Jul-10 | Aug-10 | Sep-10 | Oct-10 | Nov-10 | Dec-10 | Jan-11 | Feb-11 | Mar-11 | Apr-11 | May-11 | Jun-11 | Jul-11 | Aug-11 |
| **Clinical load** |  |  |  |  |  |  |  |  |  |  |  |  |  |  |  |  |  |  |
| recruitment total | 8 | 8 | 8 | 8 | 8 | 8 | 8 | 8 | 8 | 8 | 8 | 8 |  |  |  |  |  |  |
| recruitment to intervention | 4 | 4 | 4 | 4 | 4 | 4 | 4 | 4 | 4 | 4 | 4 | 4 |  |  |  |  |  |  |
| intervention total current | 4 | 8 | 12 | 12 | 12 | 12 | 12 | 12 | 12 | 12 | 12 | 12 | 8 | 4 |  |  |  |  |
|  |  |  |  |  |  |  |  |  |  |  |  |  |  |  |  |  |  |  |
| **Equipment load** |  |  |  |  |  |  |  |  |  |  |  |  |  |  |  |  |  |  |
| equipment in use | 4 | 8 | 12 | 12 | 12 | 12 | 12 | 12 | 12 | 12 | 12 | 12 | 8 | 4 |  |  |  |  |
|  |  |  |  |  |  |  |  |  |  |  |  |  |  |  |  |  |  |  |
| **Researcher load** |  |  |  |  |  |  |  |  |  |  |  |  |  |  |  |  |  |  |
| new patient training | 4 | 4 | 4 | 4 | 4 | 4 | 4 | 4 | 4 | 4 | 4 | 4 |  |  |  |  |  |  |
| patient baseline assessment | 8 | 8 | 8 | 8 | 8 | 8 | 8 | 8 | 8 | 8 | 8 | 8 |  |  |  |  |  |  |
| patient 3 month assessment |  |  |  | 8 | 8 | 8 | 8 | 8 | 8 | 8 | 8 | 8 | 8 | 8 | 8 |  |  |  |
| patient 6 month assessment |  |  |  |  |  |  | 8 | 8 | 8 | 8 | 8 | 8 | 8 | 8 | 8 | 8 | 8 | 8 |
| total assessments + training | 12 | 12 | 12 | 20 | 20 | 20 | 28 | 28 | 28 | 28 | 28 | 28 | 16 | 16 | 16 | 8 | 8 | 8 |
|  |  |  |  |  |  |  |  |  |  |  |  |  |  |  |  |  |  |  |
| **Total patients experiencing telecare system** | | | **48** |  |  |  |  |  |  |  |  |  |  |  |  |  |  |  |

| **Canterbury** |  |  |  |  |  |  |  |  |  |  |  |  |  |  |  |  |  |  |
| --- | --- | --- | --- | --- | --- | --- | --- | --- | --- | --- | --- | --- | --- | --- | --- | --- | --- | --- |
|  |  |  |  |  |  |  |  |  |  |  |  |  |  |  |  |  |  |  |
| Patient numbers in active and control arm, with tallies for clinical load, equipment numbers, researcher load | | | | | | | | |  |  |  |  |  |  |  |  |  |  |
| **equipment used 2 times, 6 months per patient** | | |  |  |  |  |  |  |  |  |  |  |  |  |  |  |  |  |
| recycle equipment starts Sep 10 | |  |  |  |  |  |  |  |  |  |  |  |  |  |  |  |  |  |
|  |  |  |  |  |  |  |  |  |  |  |  |  |  |  |  |  |  |  |
|  | Mar-10 | Apr-10 | May-10 | Jun-10 | Jul-10 | Aug-10 | Sep-10 | Oct-10 | Nov-10 | Dec-10 | Jan-11 | Feb-11 | Mar-11 | Apr-11 | May-11 | Jun-11 | Jul-11 | Aug-11 |
| **Clinical load** |  |  |  |  |  |  |  |  |  |  |  |  |  |  |  |  |  |  |
| recruitment total | 8 | 8 | 8 | 8 | 8 | 8 | 8 | 8 | 8 | 8 | 8 | 8 |  |  |  |  |  |  |
| recruitment to intervention | 4 | 4 | 4 | 4 | 4 | 4 | 4 | 4 | 4 | 4 | 4 | 4 |  |  |  |  |  |  |
| intervention total current | 4 | 8 | 12 | 16 | 20 | 24 | 24 | 24 | 24 | 24 | 24 | 24 | 20 | 16 | 12 | 8 | 4 |  |
|  |  |  |  |  |  |  |  |  |  |  |  |  |  |  |  |  |  |  |
| **Equipment load** |  |  |  |  |  |  |  |  |  |  |  |  |  |  |  |  |  |  |
| equipment in use | 4 | 8 | 12 | 16 | 20 | 24 | 24 | 24 | 24 | 24 | 24 | 24 | 20 | 16 | 12 | 8 | 4 |  |
|  |  |  |  |  |  |  |  |  |  |  |  |  |  |  |  |  |  |  |
| **Researcher load** |  |  |  |  |  |  |  |  |  |  |  |  |  |  |  |  |  |  |
| new patient training | 4 | 4 | 4 | 4 | 4 | 4 | 4 | 4 | 4 | 4 | 4 | 4 |  |  |  |  |  |  |
| patient baseline assessment | 8 | 8 | 8 | 8 | 8 | 8 | 8 | 8 | 8 | 8 | 8 | 8 |  |  |  |  |  |  |
| patient 3 month assessment |  |  |  | 8 | 8 | 8 | 8 | 8 | 8 | 8 | 8 | 8 | 8 | 8 | 8 |  |  |  |
| patient 6 month assessment |  |  |  |  |  |  | 8 | 8 | 8 | 8 | 8 | 8 | 8 | 8 | 8 | 8 | 8 | 8 |
| total assessments + training | 12 | 12 | 12 | 20 | 20 | 20 | 28 | 28 | 28 | 28 | 28 | 28 | 16 | 16 | 16 | 8 | 8 | 8 |
|  |  |  |  |  |  |  |  |  |  |  |  |  |  |  |  |  |  |  |
| **Total patients experiencing telecare system** | | | **48** |  |  |  |  |  |  |  |  |  |  |  |  |  |  |  |

| **Tairawhiti** |  |  |  |  |  |  |  |  |  |  |  |  |  |  |  |  |  |  |
| --- | --- | --- | --- | --- | --- | --- | --- | --- | --- | --- | --- | --- | --- | --- | --- | --- | --- | --- |
|  |  |  |  |  |  |  |  |  |  |  |  |  |  |  |  |  |  |  |
| Patient numbers in active arm, with tallies for clinical load, equipment numbers, researcher load (no control arm) | | | | | | | | |  |  |  |  |  |  |  |  |  |  |
| **equipment used 2 times, 6 months per patient** | | |  |  |  |  |  |  |  |  |  |  |  |  |  |  |  |  |
| recycle equipment starts Sep 10 | |  |  |  |  |  |  |  |  |  |  |  |  |  |  |  |  |  |
|  |  |  |  |  |  |  |  |  |  |  |  |  |  |  |  |  |  |  |
|  | Mar-10 | Apr-10 | May-10 | Jun-10 | Jul-10 | Aug-10 | Sep-10 | Oct-10 | Nov-10 | Dec-10 | Jan-11 | Feb-11 | Mar-11 | Apr-11 | May-11 | Jun-11 | Jul-11 | Aug-11 |
| **Clinical load** |  |  |  |  |  |  |  |  |  |  |  |  |  |  |  |  |  |  |
| recruitment | 2 | 2 | 2 | 2 | 2 | 2 | 2 | 2 | 2 | 2 | 2 | 2 |  |  |  |  |  |  |
|  |  |  |  |  |  |  |  |  |  |  |  |  |  |  |  |  |  |  |
| intervention total current | 2 | 4 | 6 | 8 | 10 | 12 | 12 | 12 | 12 | 12 | 12 | 12 | 10 | 8 | 6 | 4 | 2 |  |
|  |  |  |  |  |  |  |  |  |  |  |  |  |  |  |  |  |  |  |
| **Equipment load** |  |  |  |  |  |  |  |  |  |  |  |  |  |  |  |  |  |  |
| equipment in use | 2 | 4 | 6 | 8 | 10 | 12 | 12 | 12 | 12 | 12 | 12 | 12 | 10 | 8 | 6 | 4 | 2 |  |
|  |  |  |  |  |  |  |  |  |  |  |  |  |  |  |  |  |  |  |
| **Researcher load** |  |  |  |  |  |  |  |  |  |  |  |  |  |  |  |  |  |  |
| new patient training | 2 | 2 | 2 | 2 | 2 | 2 | 2 | 2 | 2 | 2 | 2 | 2 |  |  |  |  |  |  |
| patient baseline assessment | 2 | 2 | 2 | 2 | 2 | 2 | 2 | 2 | 2 | 2 | 2 | 2 |  |  |  |  |  |  |
| patient 3 month assessment |  |  |  | 2 | 2 | 2 | 2 | 2 | 2 | 2 | 2 | 2 | 2 | 2 | 2 |  |  |  |
| patient 6 month assessment |  |  |  |  |  |  | 2 | 2 | 2 | 2 | 2 | 2 | 2 | 2 | 2 | 2 | 2 | 2 |
| total assessments + training | 4 | 4 | 4 | 6 | 6 | 6 | 8 | 8 | 8 | 8 | 8 | 8 | 4 | 4 | 4 | 2 | 2 | 2 |
|  |  |  |  |  |  |  |  |  |  |  |  |  |  |  |  |  |  |  |
| **Total patients experiencing telecare system** | | | **24** |  |  |  |  |  |  |  |  |  |  |  |  |  |  |  |
